# CAMK1D activates AMPK/PINK1/Parkin-dependent mitophagy to promote enzalutamide resistance in prostate cancer

**DOI:** 10.1038/s41419-025-08342-0

**Published:** 2025-12-19

**Authors:** Feifei Sun, Ying Qu, Shuyu Mei, Lin Zhang, Xianhao Shao, Xinpei Wang, Shijia Liu, Meng Wang, Wenyao Liu, Muyi Yang, Jing Hu, Benkang Shi, Lin Gao, Bo Han

**Affiliations:** 1 Department of Pathology, Peking University People’s Hospital, Beijing, P. R. China; 2 Department of Urology, Qilu Hospital, Shandong University, Jinan, P. R. China; 3 Department of Pharmacy, The Second Qilu Hospital of Shandong University, Jinan, P. R. China; 4 Department of Pathology, Women and Children’s Hospital, Qingdao University, Qingdao, P. R. China; 5 Binzhou Center for Disease Control and Prevention, Binzhou, P. R. China; 6 Central Laboratory, Shandong Provincial Hospital Affiliated to Shandong First Medical University, Jinan, P. R. China; 7 Department of Pathology, National Cancer Center/National Clinical Research Center for Cancer/Cancer Hospital, Chinese Academy of Medical Sciences and Peking Union Medical College, Beijing, P. R. China; 8 Department of General Surgery, Qilu Hospital, Shandong University, Jinan, P. R. China; 9 School of Life Science, Faculty of Science, University of Technology, Sydney, NSW Australia; 10 Department of Pathology, Qilu Hospital, Shandong University, Jinan, P. R. China; 11 Department of Oncology-Pathology, Karolinska Institutet, Stockholm, Sweden; 12 Department of Biochemistry and Molecular Biology, School of Basic Medical Sciences, Cheeloo College of Medicine, Shandong University, Jinan, P. R. China

**Keywords:** Cancer stem cells, Targeted therapies

## Abstract

Although androgen receptor (AR)-targeted therapies, such as enzalutamide, initially improve outcomes of prostate cancer (PCa) patients, resistance inevitably develops, partly driven by prostate cancer stem-like cells (PCSCs). However, the molecular mechanisms linking the maintenance of PCSCs to enzalutamide resistance (ENZR) remain incompletely elucidated. Here, we implicate Ca²⁺/calmodulin-dependent protein kinase 1D (CAMK1D) in PCSC-mediated ENZR. CAMK1D was consistently upregulated in PCa with ENZR and contributed to ENZR by enhancing mitophagy in PCa cells both in vitro and in vivo. Mechanistically, CAMK1D promotes the expansion of PCSCs by enhancing mitophagy through activation of the AMP-activated protein kinase (AMPK)/PINK1 signaling pathway, thereby facilitating cellular adaptation. We revealed that CAMK1D interacts with and phosphorylates AMPK at Thr172, which in turn activates PINK1 to modulate mitophagy, ultimately supporting the expansion of PCSCs under enzalutamide treatment. In a mouse orthotopic PCa model, targeting the CAMK1D/AMPK pathway with the siCAM/HLNP nanoformulation suppresses tumor growth by depleting the PCSCs population, achieving a synergistic effect with enzalutamide therapy. Our findings identify CAMK1D as a key regulator of ENZR that maintains stemness by orchestrating mitophagy, thereby establishing mitophagy as an important nexus between CAMK1D-mediated ENZR and AMPK-driven PCSC enrichment. Therapeutically, we developed a CAMK1D-targeted approach that potently reverses ENZR and improves treatment responses.

## Introduction

Prostate cancer (PCa) represents the second most frequently diagnosed malignancy and fifth leading cause of cancer-related mortality among men globally [1]. Androgen receptor (AR) pathway inhibitors, including enzalutamide, have emerged as foundational therapies for advanced PCa, either as monotherapy in castration-resistant disease or in combination with androgen deprivation therapy (ADT) for hormone-sensitive cases.

Mounting evidence implicates prostate cancer stem-like cells (PCSCs) as pivotal mediators of resistance to enzalutamide. These cells exhibit hallmark stemness traits -including aberrant expression of markers such as CD44, CD133, ALDH1A1, and Nanog, alongside enhanced self-renewal capacity and lineage plasticity, collectively fostering intratumoral heterogeneity and therapeutic escape [2–4]. Notably, PCSCs engage diverse resistance mechanisms: sustaining a therapy-resistant cellular reservoir, facilitating neuroendocrine trans-differentiation, and repopulating tumors bulk through proliferative progeny, ultimately propelling disease progression to castration-resistant states [5, 6]. Therefore, targeting PCSCs holds promise for overcoming enzalutamide resistance (ENZR) in PCa and enhancing antitumor efficacy.

Ca²⁺/calmodulin-dependent protein kinase 1D (CAMK1D), a member of the Ca²⁺/calmodulin-dependent kinase family, orchestrates key cellular processes including proliferation, epithelial-mesenchymal transition (EMT), and survival [7–9]. Emerging evidence positions CAMK1D as a central mediator of calcium-mitochondrial signaling, a pathway integral to stem cell division and fate specification [10]. Recent investigations have highlighted the involvement of CAMK1D in cancer, particularly in the context of therapeutic resistance [7]. In PCa, CAMK1D overexpression drives androgen-independent growth, whereas genetic silencing impairs the proliferation and migration of LNCaP cells [11]. Moreover, Volpin et al. demonstrated that cytotoxic T lymphocytes-induced activation of CAMK1D via Fas receptor signaling accelerates tumor progression by phosphorylating and inhibiting executioner caspase-3, -6, and -7 [7]. Intriguingly, James et al. demonstrated that CAMK1D overexpression attenuates angiogenesis [12], suggesting a dualistic role-simultaneously enhancing tumor survival and altering vascular normalization, thereby modulating the tumor immune-microenvironment to potentiate immunotherapeutic responsiveness [13].

In this study, we identified CAMK1D-high PCSCs as the clonogenic subpopulation responsible for ENZR. To specifically target these therapy-resistant cells, we engineered a CD44-targeted lipid-polymer-based nanoactivator (siCAM/HLNP) for the efficient delivery of CAMK1D targeting siRNA. The siCAM/HLNP platform demonstrated that effective depletion of CAMK1D expression significantly impacted the self-renewal capacity of ENZR cells via mitophagy inhibition. The therapeutic efficacy of siCAM/HLNP was validated across multiple preclinical models, including ENZR cell lines, orthotopic resistance models, and patient-derived organoids. Collectively, these findings establish siCAM/HLNP as a promising precision therapy for stemness-mediated drug resistance in advanced PCa.

## Methods

### PCa samples

This study included 226 PCa patients diagnosed at Qilu Hospital between 2010 and 2023. Of these, 184 patients with localized cases underwent radical prostatectomy, while 42 patients with CRPC cases were managed via transurethral resection. All human tissue samples used in this study were obtained with informed consent from all participants. Histopathological evaluation was independently performed by two pathologists (J.H. and B.H.). The study was approved by the Institutional Review Board of Shandong University (Document No. KYll-2022(ZM)-1359, date: January 1, 2024) and conducted following international ethical guidelines.

### Cell lines

Cell lines LNCaP (CVCL_0395), C4-2B (CVCL_4784), RM-1 (CVCL_B459), and HEK293T (CVCL_0063) were obtained from the American Type Culture Collection (ATCC) and cultured following ATCC’s instructions. The LR (LNCaP-ENZR) and CR (C4-2B-ENZR) cell lines were established as previously described [14] and were cultured in the presence of 10 µM enzalutamide (Energy Chemical, catalog no. E120461, Anhui, China). In vivo experiments were conducted using the naturally ENZR mouse PCa cell line RM-1 to explore resistance mechanisms and therapeutic responses [15]. All cell lines were routinely tested for mycoplasma contamination and confirmed negative (Beyotime, catalog no. C0298S, Shanghai, China). To ensure experimental consistency, the cumulative culture duration from thawing to experimental use did not exceed 15 passages.

### Mitochondrial activity assessment

Mitochondrial activity was measured using MitoTracker^TM^ Deep Red FM (Thermo Fisher Scientific, catalog no. A66472, MA, USA) and JC-1 Mitochondrial Membrane Potential Assay Kit (Beyotime, catalog no. C2006, Shanghai, China). Briefly, these cells were incubated with MitoTracker or JC-1 for 20 min at 37°C. Alternatively, PCSCs were immobilized on coverslips precoated with matrix gel and subsequently incubated with MitoTracker and Hoechst for 30 min at 37 °C, followed by a fluorescence microscopy analysis.

### In vitro kinase assay

Purified recombinant 6xHis-AMPK (500 ng) was incubated with or without purified recombinant GST-CAMK1D (500 ng) in a 50 μL reaction mixture containing kinase buffer (20 mM MOPS, pH 7.2, 25 mM β-glycerol phosphate, 1 mM sodium orthovanadate, 1 mM dithiothreitol), 500 μM ATP, and 15 mM MgCl₂ at 25 °C for 1 h. The reaction was terminated by adding SDS-PAGE loading buffer and heating to 100 °C for 5 min. Samples were then analyzed by SDS-PAGE.

### Patient-derived organoids

Patient-derived PCa tissues were collected and stored in preservation solution (Biogenous, catalog no. K601005, Zhejiang, China) at 4 °C and processed within 24 h. After rinsing three times with D-PBS containing penicillin-streptomycin, fat and muscle were removed, and epithelial-enriched fragments were minced into 1-3 mm³ pieces. Tissues were digested in 5% collagenase (Biogenous, catalog no. K601003, Zhejiang, China) at 37 °C (15-90 min depending on specimen size). Digestion was terminated with 2% FBS, followed by filtration through a 100 µm strainer and centrifugation at 300×*g* for 3 min at 4 °C. Red blood cell lysis was performed if necessary. Cell pellets were washed with basal medium (Biogenous, catalog no. B213152, Zhejiang, China), resuspended in cold Matrigel (Corning, catalog no. 356255, New York, USA), and plated as 40 µL domes in 24-well plates. After polymerization at 37 °C for 25 min, domes were overlaid with 500 µL prewarmed complete prostate organoid medium (MasterAim, catalog no. 10-100-072, Hangzhou, China), which was replenished every 3-4 days. Organoids reached ~100 µm in diameter within 7-10 days and were subsequently passaged as required.

### Animal models

Six-week-old male mice were purchased from Weitonglihua Biotechnology (Beijing, China). All animal experiments were conducted in strict accordance with the welfare and ethical guidelines established by the Experimental Animal Welfare and Ethics Committee of Qilu Hospital, Shandong University (Document No. KYll-2022(ZM)-1359, date: January 1, 2024). To investigate the in vivo function of CAMK1D, we performed both subcutaneous and orthotopic prostate tumorigenesis assays in mice.

LR and CR cells with stable CAMK1D knockdown were injected subcutaneously into the axillary region of nude mice at a concentration of 2 × 10^7^ cells per mouse. RM-1 cells were orthotopically injected into the prostate tissue of 5-week-old C57BL/6 mice at a concentration of 2 × 10^5^ cells per mouse. The mice were randomly divided into four groups: control (DMSO, oral gavage), enzalutamide (10 mg/kg, oral gavage), siCAMK1D/CD44-targeted lipid-based nanoparticle (siCAM/HLNP) (intravenous), and enzalutamide (10 mg/kg, oral gavage) combined with siCAM/HLNP (intravenous). In the orthotopic prostate tumor model, treatment began on day 3 after cell implantation, and the drugs were administered daily. Throughout the experiments, subcutaneous tumors were monitored to ensure they did not exceed a maximum diameter of 15 mm. Mice bearing orthotopic tumors were maintained in good condition, and body weight was assessed weekly.

### Statistical analysis

The experiments were independently conducted in triplicate or more, with reproducible outcomes across replicates. Data were presented as mean ± standard deviation (SD). Normality of continuous variables was assessed visually via histograms and boxplots. Categorical variables were presented as frequencies and percentages. For comparisons of normally distributed data, Student’s *t*-test was used; otherwise, the Mann-Whitney *U*-test was applied. Survival analysis was performed using the Kaplan-Meier method, and statistical significance was determined by the log-rank test. Statistical analyses were conducted with GraphPad Prism 10 software (RRID: SCR_002798). Associations between categorical variables were evaluated using either the Chi-Square Test or Fisher’s exact test, as appropriate. Tumor growth comparisons among groups were analyzed by analysis of variance, with significance set at *P* < 0.05.

## Results

### Enrichment of CAMK1D-high stem-like cells in PCa-ENZR

**Fig. 1:**
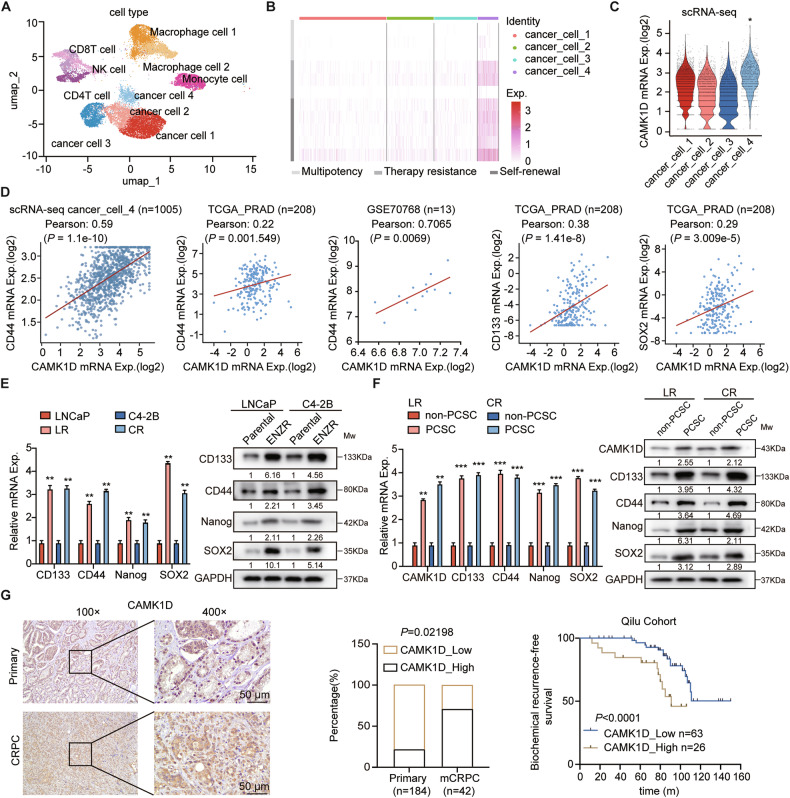
CAMK1D correlates with the regulation of stemness in PCa. **A** Integrative scRNA-seq analysis, visualized using a unified UMAP embedding for cell annotation. **B** The heatmap of multipotency, therapy resistance and self-renewal genes in cancer_cell clusters. **C** CAMK1D expression in different cancer_cell clusters. **D** CAMK1D expression positively correlated with CD44 in scRNA-seq cancer cell cluster 4, The Cancer Genome Atlas (TCGA) PCa cohort and the GSE70768 dataset, and with CD133 and SOX2 in the TCGA dataset. **E** Comparative analysis of stemness markers (CD44, CD133, Nanog, and SOX2) in ENZR versus parental cells at mRNA (RT-qPCR) and protein (western blot) levels. **F** CD44 was used to sort LR and CR cells, with CD44⁺ cells defined as PCSCs and CD44⁻ cells defined as non-PCSCs. Quantification of CAMK1D mRNA and protein levels in sorted PCSCs and non-PCSCs populations, showing enrichment of CAMK1D in PCSCs. **G** Immunohistochemistry (IHC) analysis of CAMK1D in human PCa tissues (*n* = 226) with representative images and quantification of expression. Kaplan-Meier analysis was performed to evaluate progression-free survival in patients stratified by CAMK1D expression. Data were presented as mean ± SD. Statistical significance was determined using Student’s *t*-test or log-rank test, as appropriate. LR: LNCaP-ENZR; CR: C4-2B-ENZR. **P < 0.01, ***P < 0.001.

To characterize the genomic alterations induced by enzalutamide in PCa, we established an RM-1 murine PCa allograft model in C57BL/6 mice. Tumors from both enzalutamide-treated and control groups were subjected to single-cell RNA sequencing (scRNA-seq) (Fig. 1A). The analysis revealed a significant enrichment of a stem-like cell cluster in enzalutamide-treated tumors, characterized by upregulated expression of genes associated with therapy resistance and self-renewal (Fig. 1B). To explore the link between stemness and ENZR, we intersected the top 500 genes from the stemness-related cluster 4 in our scRNA-seq data with genes showing a fold change >2 in public datasets of LR, CR, and LR xenografts. Subsequent validation in our resistant cell lines identified CAMK1D as one of the most prominently upregulated genes (Fig. 1C and Supplementary Fig. 1A, B). CAMK1D exhibited strong co-expression with canonical stemness markers across multiple datasets (scRNA-seq_CD44, Pearson *r* = 0.59, *P* = 1.1e-10; TCGA_CD44, Pearson *r* = 0.22, *P* = 0.001549; GSE70768_CD44, Pearson *r* = 0.70656, *P* = 0.0069; TCGA_CD133, Pearson r = 0.38, *P* = 1.41e-8; TCGA_SOX2, Pearson *r* = 0.29, *P* = 3.009e-5) (Fig. 1D). Consistent with scRNA-seq data, validation in previously established ENZR cell lines (LR and CR) confirmed elevated expression of stemness markers (CD44, Nanog, SOX2, and CD133) at both mRNA and protein levels compared to parental cells (Fig. 1E), indicating an enhanced stem-like phenotype in ENZR cells. CD44 was used as a marker to sort PCSCs from LR and CR cells, these PCSCs exhibited elevated CAMK1D expression (Fig. 1F). Clinical analysis of CAMK1D by immunohistochemistry from 184 primary PCa and 42 CRPC patient tissues showed significantly higher CAMK1D expression in CRPC. Critically, patients with CAMK1D overexpression demonstrated shorter disease-free duration (Fig. 1G). In summary, these results collectively demonstrate that CAMK1D is closely associated with stem cell-like properties in PCa.

### CAMK1D sustains a stem-like phenotype in ENZR cells

**Fig. 2:**
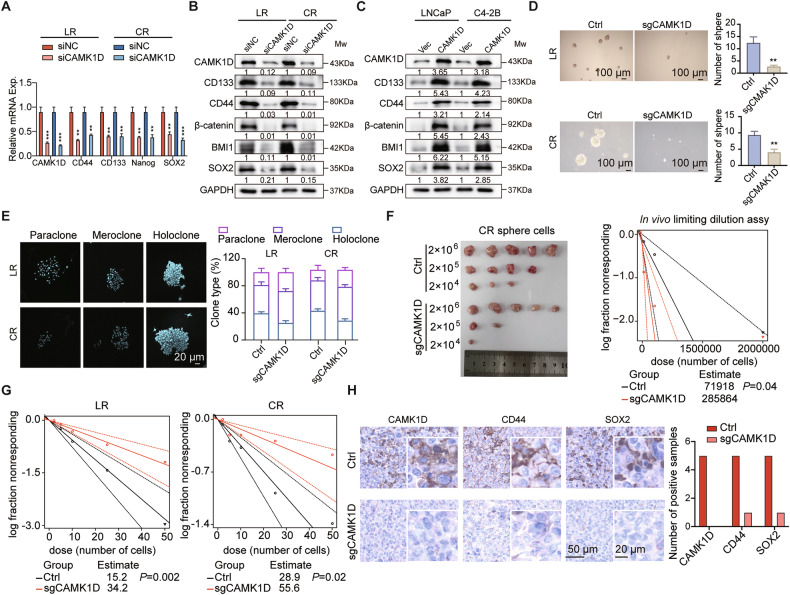
CAMK1D sustains stem-like properties in enzalutamide-resistant PCa cells. **A**–**C** Stemness markers (e.g., CD44 and CD133) expression assessed by RT-qPCR and western blot following CAMK1D knockdown (using siRNA approach) or overexpression. **D** Sphere formation assay showing the number and size of tumor spheres formed by ENZR cells upon CAMK1D knockout at day 14. Representative images and quantitative analyses were shown. **E** Colony formation assay revealing the effect of CAMK1D knockdown on stem-like colony-forming ability. LR/CR cells subjected to CAMK1D knockout were seeded for colony formation. After 14 days, colonies were fixed in paraformaldehyde and stained with DAPI for nuclear labeling. Confocal microscopy images of representative holoclones and corresponding quantification were presented. **F** In vivo tumor formation in nude mice injected with sgCAMK1D-treated CR cells. CR cells were treated with sgCAMK1D and subjected to sphere formation. Sphere cells were then diluted in a gradient and injected subcutaneously into nude mice. Tumor numbers were counted after 30 days (*n* = 5 per group, *P* = 0.04). The injected cell quantity and tumor formation frequency were shown. **G** In vitro limiting dilution assay evaluating the effects of CAMK1D knockout on stemness in LR/CR cells (*n* = 16 wells per group). **H** IHC staining of CAMK1D, CD44, and SOX2 in xenograft tumor tissues from Ctrl and sgCAMK1D groups, with quantification of the number of positive cases (*n* = 5). Data were presented as mean ± SD from at least three independent experiments. Statistical significance was determined by Student’s *t*-test or extreme limiting dilution analysis. **P < 0.01, ***P < 0.001.

We next determined whether CAMK1D expression is functionally required to maintain stemness properties in ENZR cells. We first confirmed that all three genetic modalities (siRNA, shRNA and sgRNA) efficiently knocked down CAMK1D at the protein level in ENZR cell lines, as demonstrated by western blot analysis (Supplementary Fig. 2A). As shown in Fig. 2A, B, siRNA-mediated CAMK1D knockdown significantly reduced expression of stemness markers (e.g., CD44 and CD133) at both mRNA and protein levels, while CAMK1D overexpression conversely upregulated these markers (Fig. 2C). These results suggested CAMK1D as a positive regulator of stemness-associated gene expression. Functionally, CAMK1D knockout significantly impaired clonogenic potential, as evidenced by significant reduction in tumor sphere number, whereas CAMK1D overexpression enhanced the sphere-forming capacity of ENZR cells (Fig. 2D and Supplementary Fig. 2B). In parallel, CAMK1D-silenced cells exhibited a reduction in holoclone formation, which is a well-recognized hallmark of stem-like cell behavior in vitro [16], by ~20% (LR) and 18% (CR)(Fig. 2E). In vivo limiting dilution assays further substantiated the essential role of CAMK1D, showing that its silencing markedly suppressed the tumor-initiating capacity of CR sphere-forming cells (*P* = 0.04) (Fig. 2F). This reduction in stemness was corroborated by decreased sphere formation following CAMK1D knockout in both LR (*P* = 0.002) and CR (*P* = 0.02) models (Fig. 2G). Furthermore, tumors from the sgCAMK1D group exhibited substantially lower levels of CD44 and SOX2 compared to those from the Ctrl group (Fig. 2H). Collectively, these data indicate that CAMK1D maintains the stem-like properties of PCa-ENZR cells and contributes to tumor initiation both in vitro and in vivo.

### CAMK1D facilitates ENZR via expansion of stem-like cell populations

**Fig. 3:**
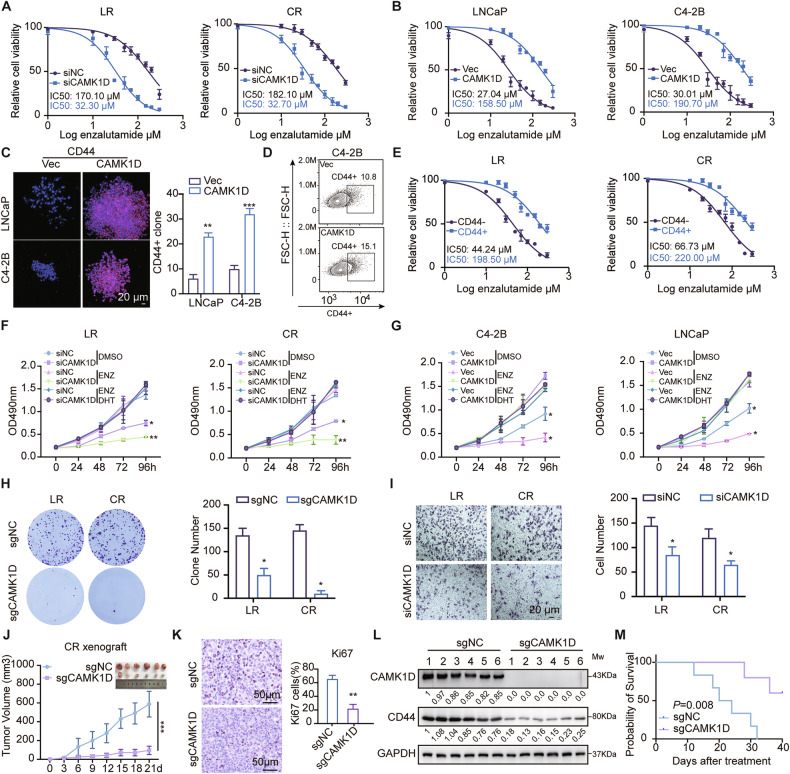
CAMK1D facilitates ENZR via expansion of stem-like cell populations. **A**, **B** Functional assays in LR/CR and LNCaP/C4-2B cells following CAMK1D knockdown (using siRNA approach) or overexpression. LR and CR cells were transfected with siCAMK1D, whereas LNCaP/C4-2B cells overexpressed CAMK1D. IC50 assays were performed under enzalutamide treatment. **C** LNCaP and C4-2B cells overexpressing CAMK1D were treated with 10 μM enzalutamide for 14 days and subjected to colony formation assays. Cells were fixed in paraformaldehyde, incubated overnight with an anti-CD44 primary antibody, and stained with a fluorescent secondary antibody. Nuclei were counterstained with DAPI and visualized by confocal microscopy. **D** C4-2B cells overexpressing CAMK1D were treated with 10 μM enzalutamide for 48 h, labeled with fluorescently conjugated anti-CD44 antibody, and analyzed by flow cytometry to determine the proportion of CD44⁺ cells. **E** LR and CR cells were sorted into CD44⁻ and CD44⁺ populations, and the IC50 values for enzalutamide were determined separately for each subset. **F**, **G** Assess cell proliferation in LR/CR and LNCaP/C4-2B cells following CAMK1D knockdown or overexpression, in the presence or absence of enzalutamide/DHT treatment. siCAMK1D was transfected into LR/CR cells, whereas CAMK1D was overexpressed in control cells (LNCaP/C4-2B). **H** Colony formation assays in LR and CR cells following CAMK1D knockout (using sgRNA approach). **I** Invasion assays in LR and CR cells following CAMK1D knockdown. **J** Tumor growth curves of subcutaneous xenografts derived from CR cells with stable CAMK1D knockout (*n* = 6/group). **K** Immunohistochemical staining of Ki67 in mouse tumor tissues. **L** Western blot analysis of CD44 expression in tumor tissues from control and CAMK1D-silenced groups. **M** Kaplan-Meier survival curve of tumor-bearing mice. Data were presented as mean ± SD from at least three independent experiments. Statistical significance was determined using Student’s *t*-test. ENZ enzalutamide.*P < 0.05, **P < 0.01, ***P < 0.001.

To investigate whether CAMK1D-induced stem-like phenotype influences to enzalutamide response, we performed loss- and gain- of function experiments under enzalutamide treatment. CAMK1D knockdown enhanced the enzalutamide sensitivity in resistant cells, whereas its overexpression increases resistance in parental cells (Fig. 3A, B). Clonogenic survival assays combined with CD44 immunofluorescence staining, demonstrated significant expansion of CD44⁺ cells in CAMK1D-overexpressing group under enzalutamide treatment (Fig. 3C). This finding was further validated by flow cytometry, which showed a higher proportion of CD44⁺ cells in the same group (Fig. 3D). Of note, CD44^+^ cells increased half-maximal inhibitory concentration (IC50) values for enzalutamide (Fig. 3E). CAMK1D also enhanced proliferative capacity of ENZR cells. Silencing CAMK1D enhanced the inhibitory effect of enzalutamide on PCa cell lines, whereas overexpressing CAMK1D attenuated this effect (Fig. 3F, G). Simultaneously, depletion of CAMK1D inhibited colony formation and invasive potential in LR and CR cells under enzalutamide treatment (Fig. 3H, I). To validate the role of CAMK1D in ENZR in vivo, we established a mouse model receiving enzalutamide treatment (Supplementary Fig. 3A). Suppressing CAMK1D in CR xenografts significantly reduced tumor growth. The mean tumor volumes were 95.71 mm³ in the knockout group and 276.6 mm³ in the knockdown group, compared to 588.08 and 780.4 mm³ in their respective controls (Fig. 3J and Supplementary Fig. 3B). The immunohistochemical staining of Ki67 revealed lower proliferation in CAMK1D-silenced tumors (Fig. 3K and Supplementary Fig. 3C), and western blot revealed reduced CD44 expression (Fig. 3L and Supplementary Fig. 3D). More importantly, mice with low CAMK1D expression exhibited markedly prolonged survival (Fig. 3M and Supplementary Fig. 3E). These observations were recapitulated in CAMK1D knockdown tumors models. Collectively, these results indicate that CAMK1D promotes the development of ENZR by expanding the stem cell-like population in PCa.

### CAMK1D enhances mitophagy in PCSCs

**Fig. 4:**
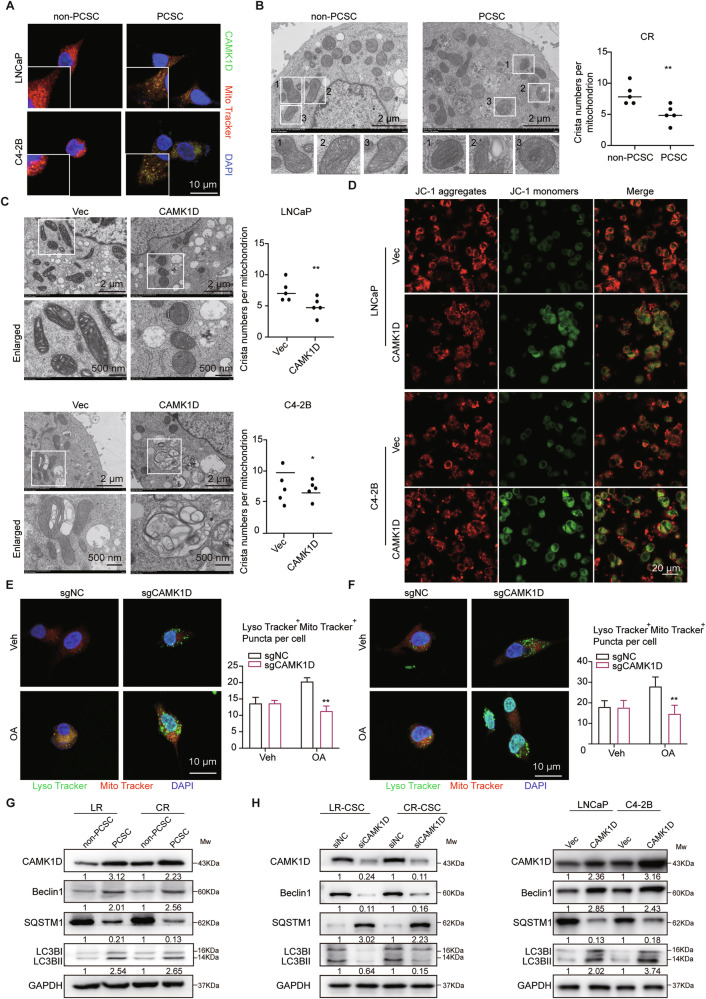
CAMK1D enhances mitophagy in PCSCs. **A** Subcellular localization of CAMK1D (red) and mitochondria (MitoTracker Green) in PCSCs visualized by confocal immunofluorescence microscopy. **B** TEM ultrastructural analysis of mitochondrial morphology in non-PCSCs and PCSCs, showing increased mitophagic vacuoles in PCSCs (The boxed region indicates a magnified view). **C** TEM visualization of mitophagic structures in LNCaP and C4-2B cells overexpressing CAMK1D. **D** Quantitative analysis of mitochondrial membrane potential by JC-1 staining in non-PCSCs LNCaP with or without CAMK1D overexpression. **E**, **F** Mitochondria-lysosome co-localization in OA-stimulated PCSCs with or without CAMK1D knockout, assessed by Pearson’s coefficient (Lyso Tracker: lysosomal marker; MitoTracker: mitochondria marker). **G** Western blot analysis of mitophagy markers (Beclin1, LC3B-II, SQSTM1) in PCSCs and non-PCSCs. **H** Western blot analysis of mitophagy markers (Beclin1, LC3B-II, SQSTM1) in PCSCs (or LNCaP/C4-2B cells), with or without CAMK1D knockdown (or overexpression). The relative expression levels of target proteins analyzed by western blot were calculated as the ratio of the grayscale intensity of the target band to that of the GAPDH band (for LC3B, quantification was based on the ratio of LC3B-II to LC3B-I, normalized to GAPDH). Data were represented as mean ± SD from at least three independent experiments. Statistical significance was determined using Student’s *t*-test. LR-CSC, LR CD44^+^; CR-CSC, CR CD44^+^. *P < 0.05, **P < 0.01.

To elucidate the metabolic characteristics of PCSCs, we first performed GSEA on genes upregulated in the PCSCs cluster relative to other clusters identified from the scRNA-seq data. This analysis revealed significant enrichment of AKT and AMPK signaling pathways, oxidative phosphorylation, and mitophagy-related processes (Supplementary Fig. 4A), suggesting that metabolic reprogramming and mitophagy may be critical features of PCSCs. Supporting this notion, immunofluorescence analysis demonstrated distinct mitochondrial localization of CAMK1D in PCSCs, as evidenced by co-staining with MitoTracker (Fig. 4A). Ultrastructural analysis by transmission electron microscopy (TEM) revealed a markedly increased number of mitophagic vacuoles with a reduction of crista numbers per mitochondrion in PCSCs compared to non-PCSCs (Fig. 4B), highlighting the enhanced mitophagic activity in the stem-like population. To functionally validate these observations, we overexpressed CAMK1D in non-PCSCs, which led to a marked increase in mitophagic structures as observed by TEM and a pronounced reduction in mitochondrial membrane potential, as assessed by JC-1 staining (Fig. 4C, D). Likewise, CAMK1D knockdown led to similar mitophagy changes in CR-CSCs with oligomycin/antimycin A (OA) treatment, a well-established inducer of mitophagy, as revealed by TEM (Supplementary Fig. 4B). In CR-CSCs, OA treatment markedly increased the levels of p-AMPK, PINK1, the stemness markers SOX2 and CD44, as well as the autophagy markers Beclin1 and SQSTM1/P62, whereas these protein changes were attenuated upon simultaneous CAMK1D silencing (Supplementary Fig. 4C). Moreover, OA stimulation induced the co-localization of mitochondria and lysosomes in PCSCs, an effect that was abrogated upon CAMK1D knockdown (LR, *P* = 0.004897; CR, *P* = 0.02097) (Fig. 4E, F). Consistently, western blot analysis confirmed that the CAMK1D-mediated regulation of mitophagy increased the expression of mitophagy-related proteins, including Beclin1 and LC3B, and downregulated SQSTM1/P62 expression in PCSCs compared to no-PCSCs (Fig. 4G). Moreover, mitophagy-related protein expression was decreased upon CAMK1D silencing and enhanced by CAMK1D overexpression (Fig. 4H and Supplementary Fig. 4D). Collectively, these results suggest that CAMK1D facilitates mitophagy in PCSCs, particularly in response to OA stimulation, thereby contributing to the maintenance of their stem-like properties.

### Mitophagy inhibitors reverse the CAMK1D-induced stem-like phenotype and ENZR

**Fig. 5:**
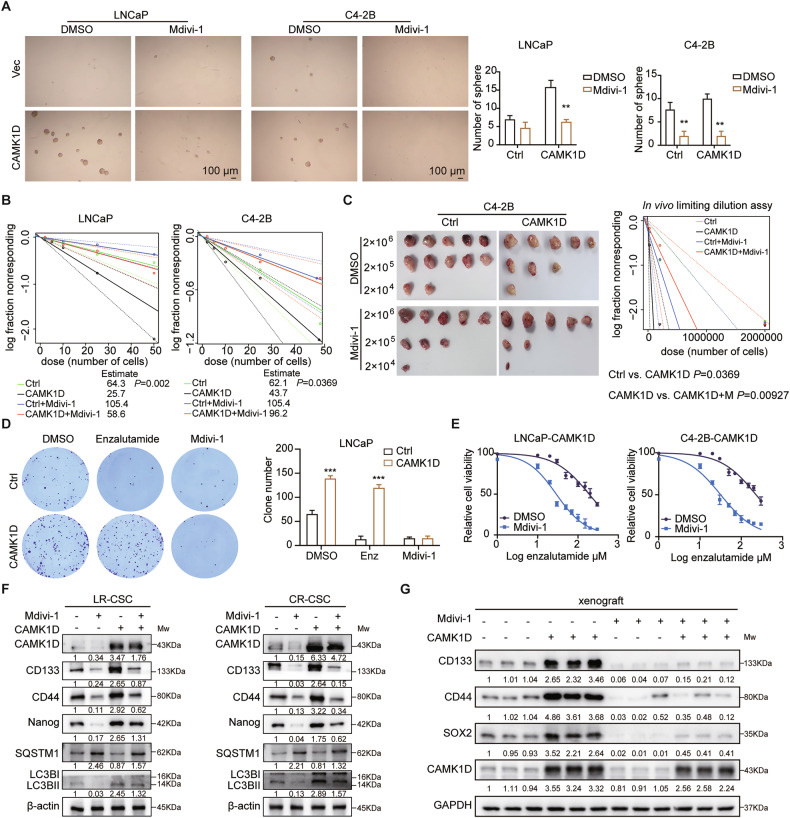
Mitophagy inhibition reverses CAMK1D-induced stem-like phenotype and ENZR. **A** Sphere formation assay in CAMK1D-overexpressing LNCaP and C4-2B cells treated with or without Mdivi-1. **B** In vitro limiting dilution assay assessing tumor-initiating capacity in CAMK1D-overexpressing cells with or without mitophagy inhibition. **C** In vivo tumor formation in nude mice injected with C4-2B cells with CAMK1D-overexpressed. C4-2B cells were serially diluted and injected subcutaneously into nude mice treated with DMSO or Mdivi-1. The number of tumors in each group were counted after 30 days. The injected cell quantity and tumor formation frequency (*n* = 5 per group, *P* = 0.00927) were shown. **D** Colony formation potential of CAMK1D-overexpressing cells treated with enzalutamide or mitophagy inhibitors. **E** IC50 assay for enzalutamide in CAMK1D-overexpressing LNCaP and C4-2B cells treated with mitophagy inhibitors. **F** Western blot analysis of CD44 and additional stemness- and mitophagy-related proteins in CAMK1D-overexpressing cells with or without mitophagy inhibitors. **G** CD44, CD133, and SOX2 expression in tumor tissues from xenograft models was assessed by Western blot to evaluate the effect of mitophagy inhibition on CAMK1D-induced stemness in vivo, with three tumors randomly selected from each group for analysis. The relative expression levels of target proteins analyzed by Western blot were calculated as the ratio of the grayscale intensity of the target band to that of the GAPDH band (for LC3B, quantification was based on the ratio of LC3B-II to LC3B-I, normalized to GAPDH). Data were represented as mean ± SD from at least three independent experiments. Statistical significance was determined using Student’s *t*-test. **P < 0.01, ***P < 0.001.

To further confirm the role of mitophagy in CAMK1D-mediated ENZR, we investigated whether pharmacological inhibition of mitophagy could reverse the effects of CAMK1D overexpression. Under enzalutamide stimulation, increased mitophagy was observed in PCa cells (Supplementary Fig. 5). Using Mdivi-1 (a selective mitophagy inhibitor that blocks mitochondrial fission and subsequent mitophagy), we demonstrated that mitophagy inhibition effectively attenuated the increase in sphere formation induced by CAMK1D overexpression in PCa cells (Fig. 5A and Supplementary Fig. 6). Similarly, the increased tumor-initiating capacity induced by CAMK1D was reversed, as demonstrated by in vitro limiting dilution assays (Fig. 5B). In vivo limiting dilution assays revealed a significant reduction in tumor-initiating capacity upon pharmacological inhibition of mitophagy in CAMK1D-overexpressing cells (Fig. 5C). Consistently, colony formation assays demonstrated that the enhanced clonogenic potential driven by CAMK1D was also significantly suppressed by mitophagy inhibitors (Fig. 5D). Notably, mitophagy inhibition restored enzalutamide sensitivity in CAMK1D-overexpressing cells, as evidenced by reduced IC50 values (Fig. 5E). Western blot analysis further revealed that the CAMK1D-induced increase in CD44 expression was abolished upon mitophagy inhibition (Fig. 5F). These findings were corroborated in xenograft models, where tumor tissues exposed to mitophagy inhibitors exhibited reduced expression of CD44 and additional stemness-related markers (Fig. 5G). Together, these findings suggest that mitophagy is indispensable for CAMK1D-induced stem-like features and therapeutic resistance in PCa.

### CAMK1D enhances mitophagy by activating AMPK/PINK1 signaling pathway in a kinase activity-dependent manner

**Fig. 6:**
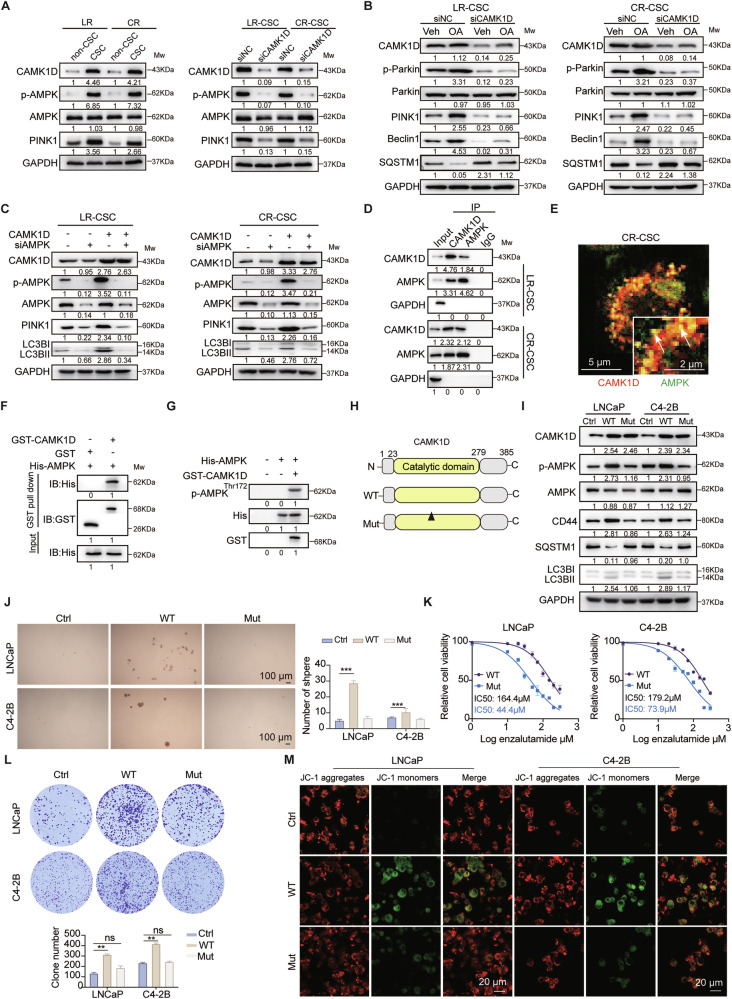
CAMK1D promotes mitophagy in PCSCs via AMPK activation. **A** Western blot analysis of AMPK and PINK1 protein expression in PCSCs and non-PCSCs populations of ENZR cells, and LR-CSC/CR-CSC cells with or without CAMK1D knockdown. **B** Western blot analysis of mitophagy markers in ENZR cells treated with OA, with or without CAMK1D knockdown. **C** Western blot analysis of mitophagy-related proteins in cells overexpressing CAMK1D, with or without AMPK knockdown. **D** Co-immunoprecipitation assays were performed in LR-CSC and CR-CSC PCa cell lines, followed by western blot analysis of the precipitated proteins. The relative expression levels of target proteins were calculated as the ratio of the grayscale intensity of the target band to that of the Input band. **E** Immunofluorescence staining of CAMK1D and AMPK in CR-CSC cells. All images were acquired using a confocal microscope. Representative images were shown with a 10 μm scale bar. **F** Direct interaction between CAMK1D and AMPK protein tested by GST pull-down assays. Purified GST-CAMK1D and His-AMPK recombinant proteins were incubated, and the complexes were analyzed by Western blot assay. The binding of target proteins was evaluated in a binary manner, with “0” indicating no binding and “1” indicating binding. **G** In vitro kinase assays were performed by mixing purified GST-CAMK1D protein with purified His-AMPK protein in the presence of ATP. **H** Schematic representation of CAMK1D protein domain structure, highlighting the catalytic domain and mutation sites in kinase-dead mutants. The binding of target proteins was evaluated in a binary manner, with “0” indicating no binding and “1” indicating binding. **I** Western blot analysis of AMPK phosphorylation, SQSTM1, LC3B, and CD44 expression in cells overexpressing wild-type or kinase-dead CAMK1D mutants. **J**–**M** Functional assays in cells expressing CAMK1D mutants, including sphere formation assay, IC50 assay under enzalutamide treatment, colony formation assay, and mitochondrial mitophagy assessment. The relative expression levels of target proteins analyzed by Western blot were calculated as the ratio of the grayscale intensity of the target band to that of the GAPDH band (for LC3B, quantification was based on the ratio of LC3B-II to LC3B-I, normalized to GAPDH). Data were represented as mean ± SD from at least three independent experiments. Statistical significance was determined using Student’s *t*-test. **P < 0.01, ***P < 0.001.

Recent studies have underscored a fundamental and evolutionarily conserved function of AMPK in safeguarding mitochondrial integrity [17]. Emerging evidence indicates that several newly identified downstream targets of AMPK are critically involved in diverse aspects of mitochondrial homeostasis, particularly in the regulation of mitophagy [18]. Our scRNA-seq analysis revealed an enrichment of AMPK signaling in PCSCs, suggesting its role in supporting mitophagy and stemness. Subsequent validation experiments in ENZR cells demonstrated that phosphorylated AMPK and PINK1 expression were markedly upregulated in PCSCs relative to non-PCSCs. Notably, knockdown of CAMK1D markedly reduced their expression (Fig. 6A). Furthermore, with OA treatment increased the levels of mitophagy-related markers including PINK1, Parkin, Beclin1, and SQSTM1/P62, but this effect was significantly diminished after CAMK1D silencing (Fig. 6B). Consistently, CAMK1D overexpression increased the levels of mitophagy-related proteins (LC3B and PINK1), whereas AMPK knockdown abolished these effects, indicating that CAMK1D promotes mitophagy via AMPK (Fig. 6C). Co-immunoprecipitation and immunofluorescence co-localization assays demonstrated an interaction between CAMK1D and AMPK (Fig. 6D, E), and GST pull-down assays further confirmed that the two proteins interact directly (Fig. 6F). In vitro kinase assays revealed that CAMK1D directly phosphorylates AMPK at Thr172 (Fig. 6G). The phosphomimetic AMPK mutant (T172D) reversed the CAMK1D knockdown-induced reduction in PINK1 and p-Parkin expression, whereas the dominant-negative AMPK mutant (T172A) produced no significant change (Supplementary Fig. 7). Collectively, these data establish that AMPK phosphorylation is vital in CAMK1D-mediated mitophagy. CAMK1D kinase activity refers to the enzyme’s ability to phosphorylate target proteins, thereby regulating cellular signaling and function. A schematic diagram was generated to illustrate the domain architecture and mutation sites (Fig. 6H). Functional assays showed that the catalytic domain mutant of CAMK1D failed to activate AMPK phosphorylation and were unable to induce changes in LC3B or stemness-associated markers such as CD44 (Fig. 6I). Importantly, these mutants had no significant effects on sphere formation, enzalutamide sensitivity, colony formation, or mitochondrial mitophagy (Fig. 6J–M). Together, these results indicate that CAMK1D enhances PCa stemness through activation of the AMPK/PINK1-dependent mitophagy pathway, and that its kinase activity is essential for this function.

### Targeted delivery of siCAMK1D and enzalutamide via CD44-nanoparticles inhibits ENZR PCa growth in preclinical models

**Fig. 7:**
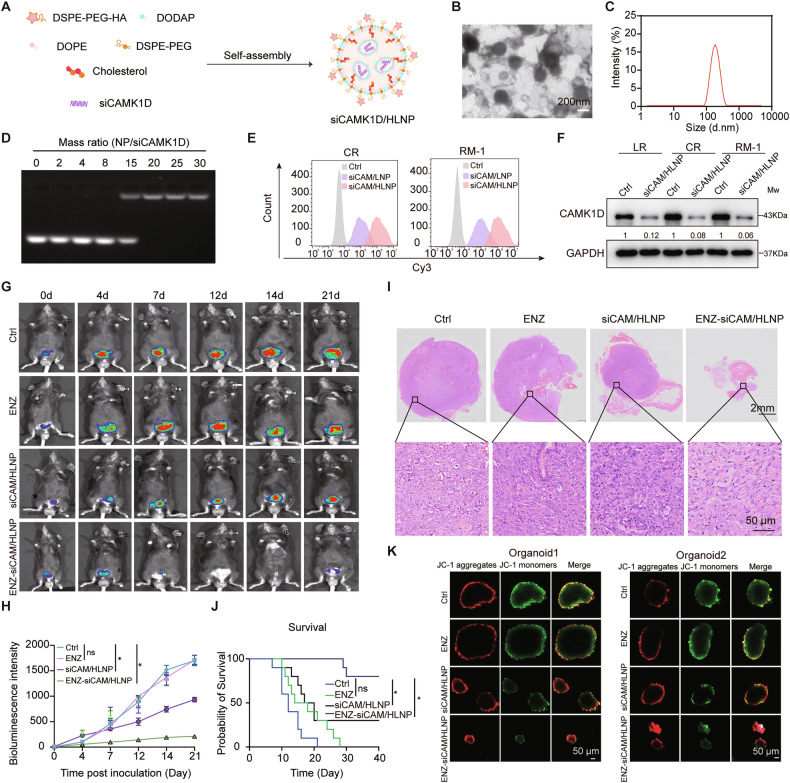
CD44-targeted nanoplatform co-delivering siCAMK1D and enzalutamide overcomes ENZR in PCa. **A** Schematic diagram illustrating the design and targeting mechanism of the CD44-directed nanoparticle loaded with siCAMK1D. **B** TEM characterization of ENZ-siCAM/HLNP nanoparticles (Scale bar, 150 nm). **C** Hydrodynamic diameter distribution determined by dynamic light scattering. **D** Agarose gel electrophoresis of siCAMK1D-NP complexes. **E** Flow cytometric analysis of cellular uptake of HLNP or LNP by the indicated PCa cells. **F** Validation of nanoparticle delivery and gene silencing efficiency in RM-1, LR and CR cells. The relative expression levels of target proteins were calculated as the ratio of the grayscale intensity of the target band to that of the GAPDH band. **G** Representative in vivo fluorescence images of tumor-bearing mice in each treatment group. **H** Quantification of tumor fluorescence intensity over time. **I** Haematoxylin and eosin staining confirming tumor histopathology. **J** Kaplan–Meier survival curves showing prolonged survival in the combination treatment group. **K** Therapeutic response of patient-derived PCa organoids to nanoparticle treatment, with immunofluorescent staining of JC-1. Data were presented as mean ± SD. Statistical significance was determined using Student’s *t*-test. *P < 0.05.

CD44 is widely expressed in CSCs across multiple tumor types [19], and its druggable extracellular domain renders it a clinically promising therapeutic target [20]. To evaluate the therapeutic potential of targeting CAMK1D-high PCSC in ENZR, we developed HLNP for delivering siCAMK1D (Fig. 7A). The nanoparticle was designed to selectively bind to PCSCs via CD44 recognition, enhancing the specificity of delivery to stem-like tumor subpopulations. Transmission electron microscopy showed that siCAM/HLNP exhibited a typical spheroid structure with an average particle size of 125.2 ± 2.3 nm (Fig. 7B). As shown in Fig. 7C, an increase in particle size upon siCAM/HLNP loading confirmed the successful preparation of siCAM/HLNP. Self-assembly at various mass ratios yielded stable siCAM/HLNP constructs, with agarose gel electrophoresis confirming stable siRNA binding to LNP at ratios above 20:1 (Fig. 7D). After 4 h of incubation, Cy3 and FITC signals were significantly higher in CR and RM-1 cells treated with siCAM/HLNP compared to those treated with siCAMK1D in lipid-based nanoparticle (siCAM/LNP), indicating enhanced cellular uptake mediated by CD44 targeting (Fig. 7E and Supplementary Fig. 8A). Western blot analysis confirmed that siCAMK1D efficiently silenced CAMK1D expression in both murine RM-1 and human ENZR cells, validating the delivery efficacy of the nanoplatform (Fig. 7F).

**Fig. 8:**
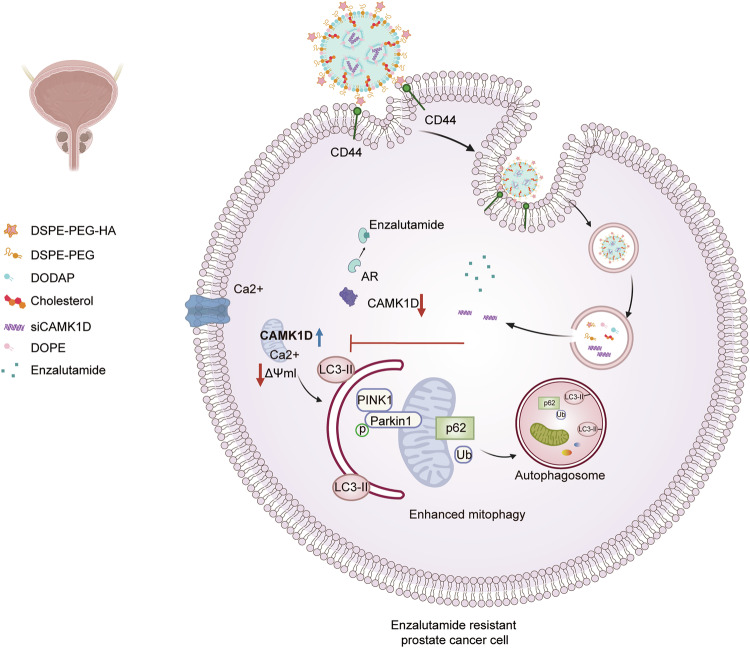
Working model of CAMK1D-induced mitophagy in promoting ENZR in PCa. The model depicts how CAMK1D-AMPK interaction triggers PINK1/Parkin-dependent mitophagy to contribute to ENZR in PCa. It also illustrates how the CD44-targeted nanoplatform co-delivers siCAMK1D and enzalutamide to simultaneously inhibit mitophagy and eliminate PCSCs, thereby suppressing tumor growth.

To evaluate the antitumor efficacy in vivo, we established orthotopic PCa models using RM-1 cells and administered the nanoparticle according to the treatment scheme shown in Supplementary Fig. 8B. In vivo imaging revealed that the Enz-siCAM/HLNP group exhibited the greatest reduction in tumor burden over time (Fig. 7G–I). Histological analysis of tumor tissues demonstrated marked reductions in tumor size and cellular proliferation (Ki67) in the combination group. TUNEL and CD44 immunofluorescence staining revealed increased apoptosis and a significant decrease in the PCSC population following treatment (Supplementary Fig. 8C, D), which translated into a significant extension in overall survival (Fig. 7J). Western blot analysis of tumor lysates confirmed downregulation of CAMK1D and CD44 expression (Supplementary Fig. 8E). RNA-seq data from the Human Protein Atlas revealed high expression of CAMK1D in the brain (Supplementary Fig. 8F). Neurotoxic effects have been reported in previous studies following CAMK1D inhibition [21, 22]. Notably, any brain injury or neurological dysfunction was not detected, highlighting the favorable safety profile and translational potential of our therapeutic approach (Supplementary Fig. 8G, H). Moreover, the therapeutic efficacy of the nanoplatform was validated in patient-derived PCa organoids, where treatment markedly reduced mitophagy levels and significantly suppressed organoid growth (Fig. 7K and Supplementary Fig. 8I). Together, these findings demonstrate that co-delivering siCAMK1D and enzalutamide via the CD44-directed nanoplatform effectively eradicates ENZR PCa cells by eliminating PCSCs and disrupting mitophagy (Fig. 8).

## Discussion

In recent years, a growing body of evidence has demonstrated PCSCs as the key mediators of tumor initiation, disease recurrence, and therapeutic resistance in PCa [23, 24]. While pivotal regulators of PCSC biology, such as TMPRSS4-mediated activation of SOX2, have been elucidated [25], the molecular circuitry underlying PCSC maintenance remains incompletely understood. Current therapeutic strategies targeting PCSCs focus on either direct pathway inhibition or microenvironmental modulation [26]. Our scRNA-seq revealed that cancer cell subcluster 4 possesses strong stemness features, with CD44 upregulated in 98% of cells. Notably, an integrated analysis intersecting its top 500 upregulated genes from this cluster with public ENZR datasets identified CAMK1D as the most distinct resistance marker, underscoring its role in PCSC maintenance and ENZR. Mechanistically, our findings establish a novel link between stemness, CAMK1D activation and AMPK-PINK1-mediated mitophagy of ENZR PCa cells, revealing that CAMK1D reinforces mitophagy in PSCSs, thereby promoting therapeutic resistance and tumor propagation.

The contribution of CSCs in driving resistance to AR-targeted therapies, including second-generation antiandrogens like enzalutamide, has become increasingly evident [27]. These cells exhibit enhanced self-renewal capacity, augmented survival advantage, and pronounced metabolic plasticity, enabling them to evade treatment-induced stress and fuel tumor progression [28]. Previous studies have shown that ADT and AR pathway inhibition can enrich for a CD44⁺CD133⁺ PCSCs population, which exhibits enhanced resistance and tumor-initiating capacity [29]. Multiple mechanisms underlie PCSC-driven endocrine resistance [1]. Activation of stemness pathways (Wnt/β-catenin, Hedgehog, Notch) sustains self-renewal and bypasses AR suppression; [2] crosstalk with tumor microenvironment components (e.g., CAFs, immune cells) enhances survival via cytokine signaling (IL-6, TGF-β) and hypoxia-induced metabolic adaptation; [3] epigenetic regulation (e.g., EZH2-mediated silencing) and cell cycle quiescence reduce therapeutic vulnerability; [4] metabolic reprogramming and expression of AR variants enable ligand-independent proliferation [30–32]. Our results align with this paradigm, demonstrating that ENZR cells exhibit elevated expression of stemness markers and increased dependence on mitochondrial quality control.

CAMK1D exerts pleiotropic regulatory effects through calcium signaling transduction, and emerging evidence implicates CAMK1D as a critical modulator of cancer progression and therapeutic resistance. Previous reports have indicated that CAMK1D can mediate metastasis via EMT activation and metabolic reprogramming [33, 34]. Here, we show that CAMK1D overexpression is associated with enhanced cellular stemness in PCa, consistent with prior evidence that CAMK1D plays a critical role in aggressive basal-like tumors with stem cell-like phenotypes [33, 35]. Our finding that CAMK1D is upregulated in PCSCs provides novel mechanistic insights into the molecular basis of therapy resistance.

Beyond their canonical roles in energy production and metabolism, mitochondria are increasingly recognized as regulators of therapeutic response [36]. In PCSCs, mitochondrial function is critical for maintaining stemness and conferring therapeutic resistance, primarily by coordinating metabolic homeostasis and autophagic signaling [37, 38]. Mitochondrial morphology and inter-organelle networking regulate the regenerative capacity of muscle stem cells by modulating cellular metabolism and proteostasis balance [39]. Mitophagy-the selective clearance of damaged or superfluous mitochondrial fragments to preserve organelle integrity-supports stemness by maintaining mitochondrial fitness and suppressing ROS accumulation. This process establishes a metabolic state conducive to self-renewal [40], prompts a quiescent metabolic profile with robust self-renewal capacity that shields stem cells from metabolic stress [41], and activates important oncogenes (e.g., c-Myc) [42].

We demonstrate that CAMK1D enhances mitophagy in PCSCs by activating AMPK phosphorylation and upregulating PINK1, thereby enabling efficient mitochondrial clearance under conditions of metabolic stress. This observation is consistent with prior evidence identifying AMPK as a central sensor of cellular energy stress that can directly regulate mitophagy via ULK1 and PINK1-dependent mechanisms [43]. Our study, therefore, bridges the gap between metabolic adaptation, stemness maintenance, and drug resistance, placing mitophagy as a functional link in this triangle.

Targeting CAMK1D via siRNA represents a promising therapeutic approach to overcome ENZR and enhance antitumor immune responses. However, the inherent limitations of siRNA, including its short half-life, suboptimal cellular uptake, rapid enzymatic degradation, and limited physicochemical stability, necessitate the development of an advanced delivery system to ensure therapeutic success. From a therapeutic perspective, targeting CSCs remains particularly challenging due to their rarity and phenotypic heterogeneity. In PCa, stemness-associated markers such as CD44, CD133 and NR4A1 have been strongly implicated in resistance to AR inhibitors, including enzalutamide [44]. CD44 is a well-established surface marker of PCSCs and has previously been investigated as a target for selective delivery systems. By designing a CD44-targeted nanoplatform capable of delivering siCAMK1D, we achieved selective accumulation within PCSCs, efficient CAMK1D silencing, and enhanced tumor suppression compared to either agent alone. Our in vivo and in vitro results provide proof-of-concept that combinatorial targeting of stemness pathways and AR signaling can effectively overcome ENZR in PCa.

In summary, this study establishes CAMK1D as a pivotal regulator of enzalutamide-resistant PCSCs maintenance and therapy resistance through its role in mediating mitophagy. Mechanistically, CAMK1D promotes mitochondrial clearance through activation of the AMPK/PINK1 signaling pathway, thereby supporting the survival and maintenance of therapy-resistant PCSCs. Therapeutically, we demonstrate that a CD44-targeted nanoplatform delivering siCAMK1D effectively eliminates PCSCs, reverses ENZR, and suppresses tumor progression in multiple preclinical models. Collectively, these findings provide both mechanistic insights and a translational rationale for targeting CAMK1D-mediated metabolic adaptations in advanced PCa.

## Supplementary information

Supplemental Materials and Methods

Supplementary tables

Supplementary Figures and Legends

Original Western blots

## Data Availability

All data and materials generated during the current study are available from the corresponding author on reasonable request.
