# Supplementary material for: CAMK1D activates AMPK/PINK1/Parkin-dependent mitophagy to promote enzalutamide resistance in prostate cancer
Source: Cell Death Dis. 2025 Dec 19;17(1):113. doi: 10.1038/s41419-025-08342-0 (PMC12847848; doi:10.1038/s41419-025-08342-0)
Supplement: Supplementary file 1 — Supplemental Materials and Methods [file 41419_2025_8342_MOESM1_ESM.docx]

**Supplementary Materials and Methods**

**Cell transfection**

The CAMK1D expression vector was synthesized by Biosune Biotech, and all siRNA sequences, along with negative controls, were provided by Origene (Human, SR311360; mouse, SR412965).

**SiRNA, sgRNA and shRNA for CAMK1D genetic manipulation**

SiRNA was chosen for its efficiency in achieving acute, transient knockdown in initial functional screens, enabling rapid phenotypic assessment; sgRNA/CRISPR-Cas9 was utilized to generate complete knockout cell lines essential for definitive functional ablation studies, while shRNA was employed for establishing stable, long-term knockdown lines required for *in vivo* models. Important experiments were performed multi-approach both transient and permanent knockdown for verification of CAMK1D's knockdown.

**Cellular viability, proliferation and clone formation assays**

Cells were seeded in 96-well plates at a density of 2,000 cells/well for LR and CR cells, with 100 μL of culture media per well. Colony formation assays were employed to evaluate cell clonogenic capacity. For clone formation assays, treated cells were seeded in 6-well plates, subjected to the respective treatments, and then fixed, stained, and counted after approximately 15 days. Oligomycin/antimycin A and Mdivi-1 were purchased from Macklin.

**Immunofluorescence staining**

Cells were cultured in confocal dishes, incubated with LysoTracker™ Red (50 nM) and MitoTracker™ Deep Red (50 nM) for 20 minutes at 37 °C, washed, fixed, and imaged by confocal microscopy. Cells were incubated with MitoTracker™ Deep Red (50 nM), fixed with 4% paraformaldehyde, permeabilized, blocked, and stained with anti-LAMP-1 antibody followed by Alexa Fluor-conjugated secondary antibody. Nuclei were counterstained with DAPI, and images were acquired using confocal microscopy.

Sphere-enriched C4-2B cancer stem-like cells were fixed with 4% paraformaldehyde for 10 minutes and permeabilized with 0.1% Tween-20 for 10 minutes. After blocking with 5% goat serum, cells were incubated overnight at 4 °C with mouse anti-CAMK1D (Santa Cruz, *sc-*374028, 1:200) and rabbit anti-AMPKα (CST, #2532, 1:200). Fluorescent secondary antibodies were applied for 1 hour in the dark, nuclei were stained with DAPI, and images were acquired using a Zeiss LSM980 confocal microscope.

Apoptosis in tumor tissues was evaluated using the TUNEL staining method according to the manufacturer's instructions (Abbkin; catalog no. ATXA30081).

**Cell migration and invasion assay**

Transwell assays were conducted to assess cellular migration and invasion. For migration, cells were transferred to 8-μm pore-size transwell inserts (Corning; catalog no. 3395). For invasion, cells were placed in Matrigel Invasion Chambers (Corning; catalog no. 354480). After 24 hours, cells in three randomly selected microscopic fields (×200) were counted for quantification.

**Western blot**

Cells or tissue samples were lysed in RIPA buffer supplemented with protease and phosphatase inhibitors. Total protein concentrations were determined using a BCA protein assay kit (Thermo Scientific). Equal amounts of protein (20-40 μg) were separated by SDS-PAGE and transferred onto PVDF membranes (Millipore). Membranes were blocked with 5% non-fat milk in TBST for 1 hour at room temperature and incubated overnight at 4 °C with primary antibodies against target protein (e.g., anti-CAMK1D, ab123456, Abcam). After washing, membranes were incubated with HRP-conjugated secondary antibodies for 1 hour at room temperature. Protein bands were visualized using enhanced chemiluminescence (ECL) reagents (Thermo Scientific) and imaged using a ChemiDoc XRS+ system (Bio-Rad). GAPDH was used as a loading control. Band intensities were quantified using ImageJ software. The details of the primary antibodies used in this study are provided in **Supplementary Table 1**.

**Co-IP**

Sphere-enriched C4-2B and LNCaP cancer stem-like cells were lysed in IP lysis buffer containing protease and phosphatase inhibitors. Cell lysates were incubated overnight at 4 °C with rabbit anti-AMPKα (CST, #2532) and mouse anti-CAMK1D (Santa Cruz, sc-374028), followed by Protein A/G beads for 2 hours. After washing, bound proteins were eluted with SDS loading buffer and analyzed by western blot to detect the interaction between AMPK and CAMK1D.

**GST-pull down**

Purified GST-CAMK1D fusion protein was immobilized on Glutathione-Sepharose 4B beads and incubated with recombinant His-AMPK protein in binding buffer (20 mM Tris-HCl, pH 7.5; 150 mM NaCl; 1 mM DTT; 0.1% NP-40) at 4 °C for 4 hours with gentle rotation. The beads were washed thoroughly to remove nonspecific binding proteins, and bound complexes were eluted with buffer containing 10 mM reduced glutathione. Eluted proteins were analyzed by SDS-PAGE followed by western blot to assess the direct interaction between CAMK1D and AMPK.

**RNA extraction and quantitative real-time PCR (RT-qPCR)**

RT-qPCR was utilized to investigate the expression levels of CAMK1D mRNA in CR and RM-1 cells following treatment with various formulations. Total RNA was extracted using Trizol reagents (Invitrogen; catalog no. 15596026) and reverse-transcribed into cDNA with the First Strand cDNA Synthesis Kit (Toyobo; catalog no. FSQ-201) according to the manufacturer's instructions. mRNA levels were quantified using a SYBR Green PCR kit (Roche; catalog no. 04887352001), with primer sequences detailed in **Supplementary Table 2**. Relative mRNA levels were calculated using the 2−ΔΔCt method, with GAPDH serving as an endogenous control.

**Immunohistochemistry (IHC)**

Tissues were fixed in 4% paraformaldehyde overnight at 4 °C, then paraffin-embedded and sectioned at a thickness of 4 μm. For histopathological assessment, sections were deparaffinized, rehydrated, and subjected to hematoxylin and eosin (H&E) staining to evaluate mucosal integrity and pathological alterations. For IHC, antigen retrieval was performed in 10 mM sodium citrate buffer (pH 6.0) at 95 °C for 10 minutes. Endogenous peroxidase activity was quenched with 3% hydrogen peroxide, followed by blocking with 5% bovine serum albumin (BSA) for 10 minutes at room temperature. The sections were then incubated overnight at 4 °C with anti-Ki67 antibody (ZM-0166, ZSGB-BIO), followed by incubation with an HRP-conjugated secondary antibody. Signal detection was achieved using DAB substrate, and nuclei were counterstained with hematoxylin. All stained sections were examined under a light microscope to assess cellular proliferation. The IHC slides were blindly evaluated by two pathologists (J.H. and B.H.) to validate the expression of CAMK1D, CD44, SOX2, and Ki67.

**Single cell RNA-seq (scRNA-seq) analysis**

An orthotopic PCa model was established in mice using RM-1 cells. Tumor tissues from control and enzalutamide-treated groups were collected for scRNA-seq. After performing quality control on the raw data, we proceeded with normalization to ensure comparability across cells. Dimensionality reduction was conducted using UMAP, facilitating visualization of cellular heterogeneity. Subsequently, we conducted cell type clustering to categorize distinct cellular populations. We analyzed differentially expressed genes (DEGs) between PCa tumor cells and related immune cells, followed by Gene Ontology (GO) and Kyoto Encyclopedia of Genes and Genomes (KEGG) enrichment analyses to elucidate the biological pathways and functions.

**Construction of siCAM/HLNP**

The acidic aqueous phase (50 mM citrate buffer, pH 4.0) containing siCAMK1D was preheated to room temperature and placed under constant stirring. Separately, an ethanol solution containing a lipid mixture-1,2-dioleoyl-3-dimethylammonium-propane (DODAP), 1,2-dioleoyl-sn-glycero-3-phosphoethanolamine (DOPE), cholesterol, DSPE-PEG-HA, and DSPE-PEG-was prepared at a defined molar ratio. The ethanol phase was rapidly injected into the aqueous siCAMK1D phase under vigorous stirring using a syringe, leading to spontaneous self-assembly of lipid nanoparticles. The RNA-to-DODAP weight ratio was optimized to ensure efficient siRNA encapsulation. The resulting sHLNPs were subjected to ultrafiltration using centrifugal filter units (MWCO 10-30 kDa) at 4 °C to remove residual ethanol and unencapsulated siRNA. The retentate was washed three times with PBS (pH 7.4) to obtain purified sHLNPs. Control LNPs (sLNPs) were prepared following the same procedure without the addition of DSPE-PEG-HA.

**Transmission electron microscopy**

The cells were collected and fixed with 2.5% glutaraldehyde at 4 °C. After pre-embedding in agarose, the cells were fixed with 1% osmium tetroxide and dehydrated with increasing ethanol concentrations. Resin blocks were prepared by embedding cells in EMBed 812 medium followed by a polymerization process. Before imaging, ultrathin sections were prepared using an ultramicrotome and stained with uranyl acetate and lead citrate. Images were captured with a transmission electron microscope (HITACHI) and analyzed with ImageJ software.

**Matrigel 3D culture**

Dissociated cells were cultured in 1640 medium with 1:50 B-27(Gibco; catalog no. A3582801), 20 ng/mL bFGF (Corning; catalog no. 354060), and 40 ng/mL EGF (Corning; catalog no. 354052). Matrigel beds were prepared in 6-well plates by placing four 50 μL drops of Matrigel per well. After incubating the plates at 37°C for 30 minutes to allow the Matrigel to solidify, 100 μL of cell suspension was mixed with 100 μL of cold Matrigel and pipetted onto the Matrigel bed. The plates were then incubated at 37°C for another 30 minutes, followed by the addition of 2.5 mL of warm 1640 per well. The cells were cultured for 10-14 days, with a 50% medium change every three days.

***In vitro* limiting dilution assay**

Cells were digested into single-cell suspensions and plated into ultra-low attachment 96-well plates in serum-free stem cell medium supplemented with growth factors. Serial dilutions (2-50 cells/well) were prepared with 16 replicates per dose. After 10-14 days, wells containing at least one tumorsphere (≥50µm) were scored as positive. Sphere-initiating cell frequency and 95% CIs were estimated using the online Extreme Limiting Dilution Analysis (ELDA) tool following the provided guidelines, with group comparisons by likelihood-ratio test and assessment of goodness-of-fit. Experiments were performed in ≥3 independent runs.

***In vivo* limiting dilution assay**

To evaluate the stem cell-like properties of ENZR cells, an *in vivo* limiting dilution assay was conducted. Briefly, following appropriate treatments, CR or C4-2B cells were dissociated into single cells and resuspended in PBS at final concentrations of 2×10⁵, 2×10⁶, and 2×10⁷ cells/mL. Subsequently, 100 μL of each cell suspension was subcutaneously injected into immunocompromised mice (n=5 per group). After 30 days, the mice were humanely euthanized, and the resulting tumors were harvested. Tumor-initiating frequency was calculated using the online ELDA tool following the provided guidelines.
