# Supplementary material for: CAMK1D activates AMPK/PINK1/Parkin-dependent mitophagy to promote enzalutamide resistance in prostate cancer
Source: Cell Death Dis. 2025 Dec 19;17(1):113. doi: 10.1038/s41419-025-08342-0 (PMC12847848; doi:10.1038/s41419-025-08342-0)
Supplement: Supplementary file 2 — Supplementary tables [file 41419_2025_8342_MOESM2_ESM.docx]

**Supplementary Table 1. Antibodies used in this study.**

| **Antibody** | **Cat no.** |
| --- | --- |
| GAPDH | ab181602, Abcam |
| CAMK1D (WB) | ab172618, Abcam |
| CAMK1D (IF, IHC) | ab198165, Abcam |
| CD44 | sc-7297, SANTA |
| CD133 | 18470-1-AP, Proteintech |
| SOX2 | ab97959, Abcam |
| Nanog | M009657S, Abmart |
| BMI1 | 6964T, Cell Signaling Technology |
| β-catenin | 8480S, Cell Signaling Technology |
| PINK1 | MA9490, Abmart |
| p-AMPK | 50081, Cell Signaling Technology |
| AMPK | 2532, Cell Signaling Technology |
| p-Parkin | 36866, Cell Signaling Technology |
| Parkin | 2132, Cell Signaling Technology |
| LAMP-1 | 9091, Cell Signaling Technology |
| Beclin1 | T55092, Abmart |
| SQSTM1 | T55546, Abmart |
| LC3B | T55992, Abmart |

**Supplementary Table 2. Sequence of primers used in this study.**

| **Gene** | **Forward** | **Reverse** |
| --- | --- | --- |
| CAMK1D | GTCCACAGAGACCTCAAGCC | GTTTCTGGGCGAGGACTTCA |
| BCL-2 | GGTGAACTGGGGGAGGATTGT | AGAGACAGCCAGGAGAAATCAAAC |
| SOX2 | GCTACAGCATGATGCAGGACCA | TCTGCGAGCTGGTCATGGAGTT |
| OCT4 | CCTGAAGCAGAAGAGGATCACC | AAAGCGGCAGATGGTCGTTTGG |
| CD44 | CCAGAAGGAACAGTGGTTTGGC | ACTGTCCTCTGGGCTTGGTGTT |
| CD133 | CACTACCAAGGACAAGGCGTTC | CAACGCCTCTTTGGTCTCCTTG |
| Nanog | CTCCAACATCCTGAACCTCAGC | CGTCACACCATTGCTATTCTTCG |
| GAPDH | GTCTCCTCTGACTTCAACAGCG | GTCTCCTCTGACTTCAACAGCG |

These primers were purchased from Generalbiol (Anhui, China).
