# Supplementary material for: CAMK1D activates AMPK/PINK1/Parkin-dependent mitophagy to promote enzalutamide resistance in prostate cancer
Source: Cell Death Dis. 2025 Dec 19;17(1):113. doi: 10.1038/s41419-025-08342-0 (PMC12847848; doi:10.1038/s41419-025-08342-0)
Supplement: Supplementary file 3 — Supplementary Figures and Legends [file 41419_2025_8342_MOESM3_ESM.docx]

**Supplementary Figures and Legends**


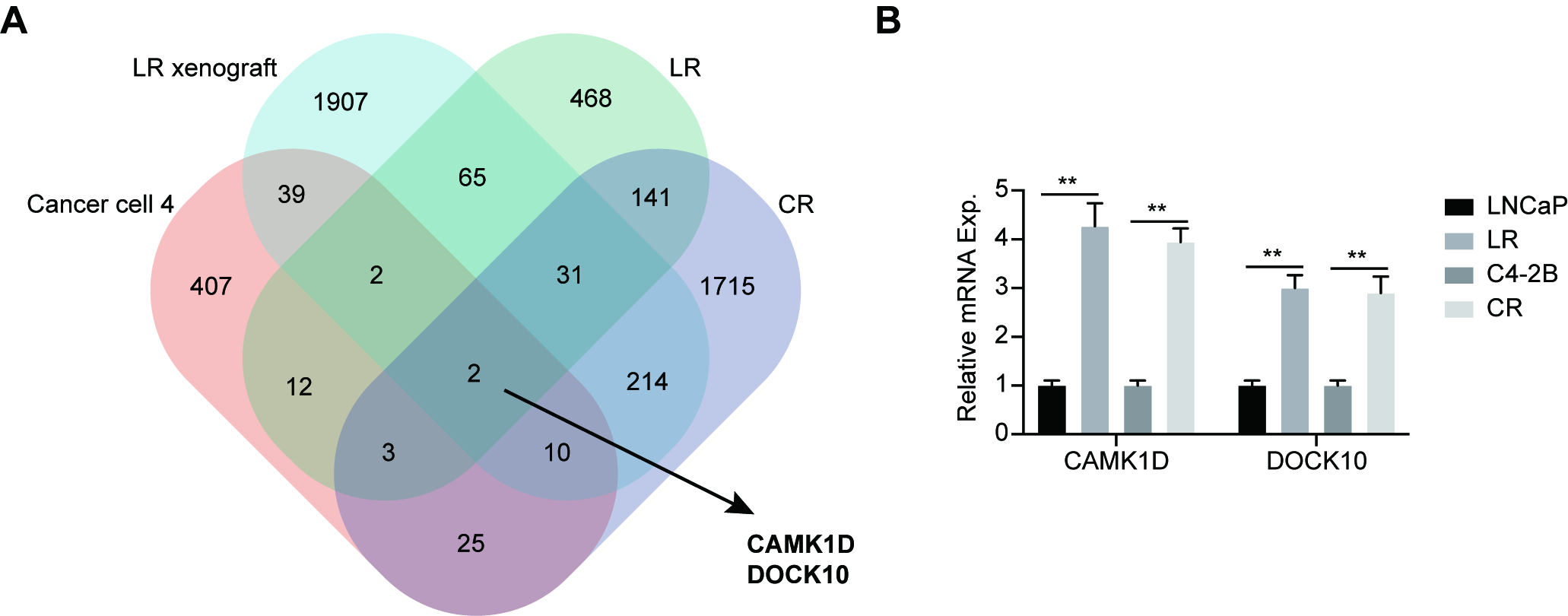


**Supplementary Fig. 1: Identification and validation of CAMK1D as a key gene associated with ENZR. A** Analysis of ENZR upregulated genes in public ENZR datasets (GSE110802 CR, GSE55345 LR xenograft, and GSE137833 LR) combined with scRNA-seq of cancer cell subcluster 4 characterized by stemness features. **B** Validation of two candidate genes in previously established enzalutamide-sensitive and -resistant cells. mRNA expression levels were quantified using RT-qPCR. LR: LNCaP-ENZR; CR: C4-2B-ENZR.

**
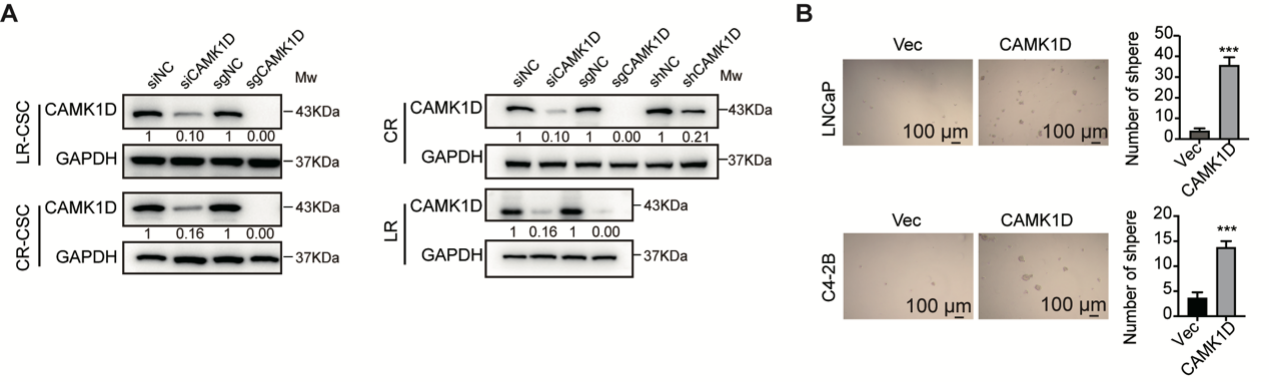
**

**Supplementary Fig. 2: CAMK1D preserves the stemness of enzalutamide-resistant PCa cells. A** Western blot analysis of CAMK1D protein expression 48 hours after transfection with siCAMK1D, shCAMK1D, or sgCAMK1D in LR-CSC, CR-CSC, CR and LR cell lines. **B** Sphere formation assay showing the number and size of tumor spheres formed by PCa cells upon CAMK1D overexpression. Representative images and quantitative analysis were shown.


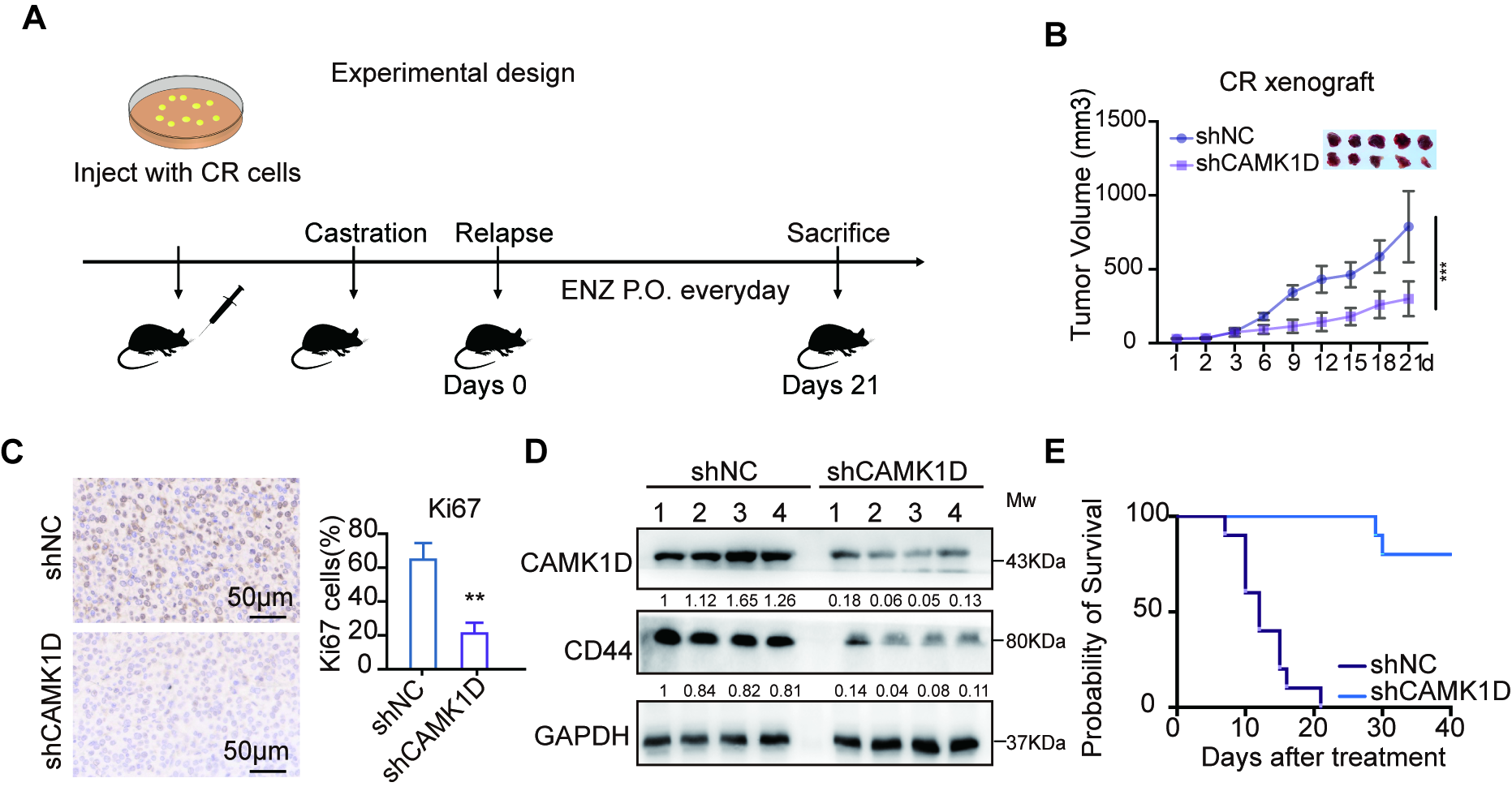


**Supplementary Fig. 3: CAMK1D knockdown significantly suppresses tumor growth *in vivo*. A** Experimental timeline and treatment protocol for *in vivo* orthotopic tumor model. **B** Tumor growth curve of CR cells with stable CAMK1D knockdown after subcutaneous injection into nude mice. **C** Immunohistochemical staining of Ki-67 in mouse tumor tissues. **D** Western blot analysis of CD44 expression in tumor tissues from control and CAMK1D-silenced groups. Due to one mouse in the experimental group having an exceptionally small tumor, protein samples from this group were incomplete. The relative expression levels of target proteins were calculated as the ratio of the grayscale intensity of the target band to that of the GAPDH band. **E** Kaplan-Meier survival curve of tumor-bearing mice.


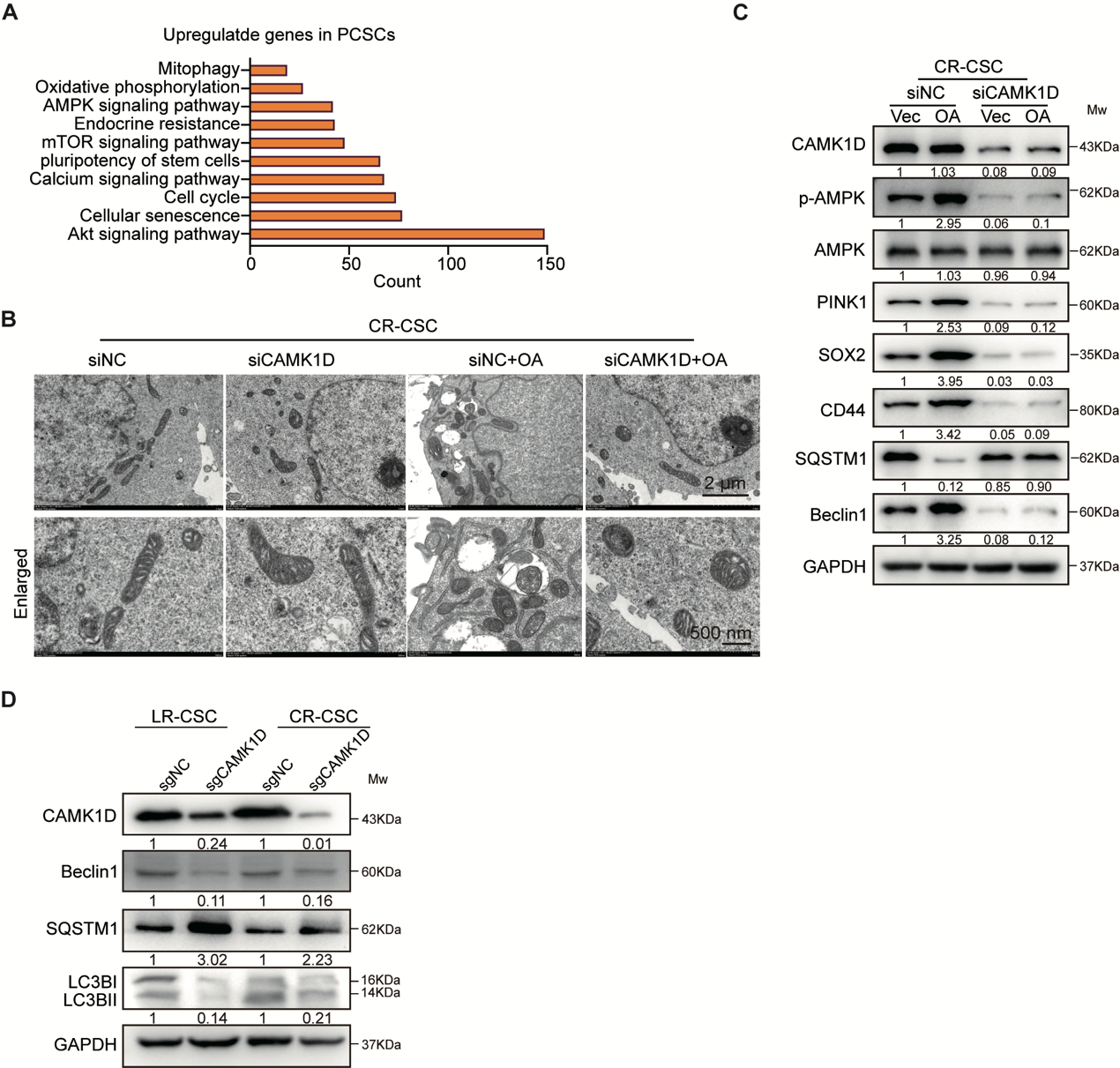


**Supplementary Fig. 4: CAMK1D regulates mitophagy in PCSCs. A** PCSCs show activated AKT/AMPK signaling and enhanced oxidative phosphorylation and mitophagy. Gene set enrichment analysis of the PCSC cluster derived from scRNA-seq data showing activation of pathways related to AKT signaling, AMPK signaling, oxidative phosphorylation, and mitophagy. **B** TEM visualization of mitochondrial/mitophagic structures in CR-CSCs following CAMK1D silencing, OA stimulation, or combined CAMK1D silencing plus OA for 24 hours. **C** Western blot analysis of mitophagy and stem marker Beclin1, SQSTM1, AMPK, PINK1, SOX2 and CD44 along with its phosphorylation level in CR-CSCs under CAMK1D silencing, OA stimulation, or combined CAMK1D silencing plus OA treatment for 24 hours. **D** Western blot analysis of mitophagy markers (Beclin1, LC3B, SQSTM1) in PCSCs and non-PCSCs, with or without CAMK1D knockout. The relative expression levels of target proteins were calculated as the ratio of the grayscale intensity of the target band to that of the GAPDH band (for LC3B, quantification was based on the ratio of LC3B-II to LC3B-I, normalized to GAPDH).


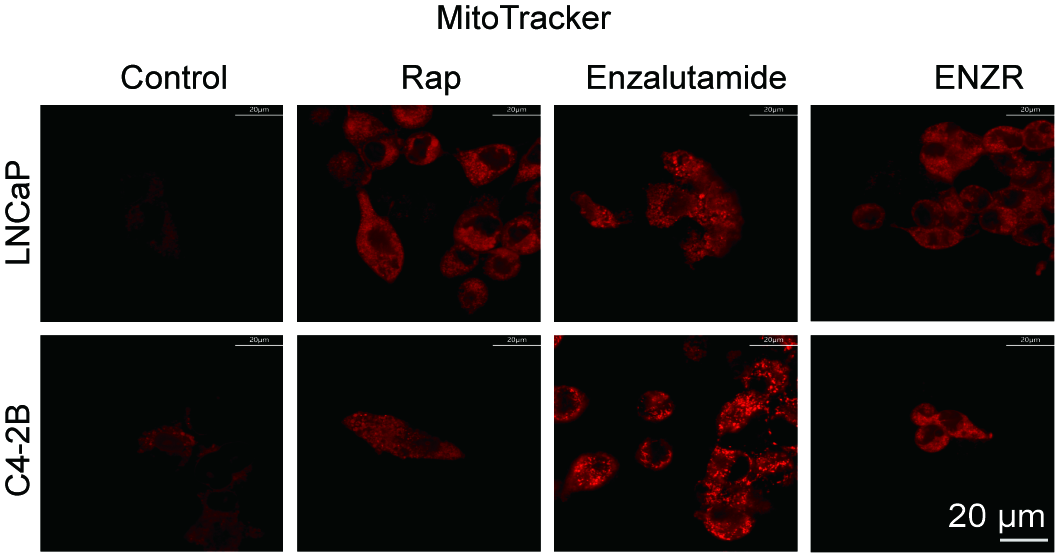


**Supplementary Fig. 5: Enzalutamide induces mitophagy and morphological differences between stem-like and non-stem-like PCa cells.** Assessment of mitochondrial levels in PCa cells treated with enzalutamide and in ENZR cell lines. Rapamycin (Rap) treatment was used as a positive control for mitophagy induction.


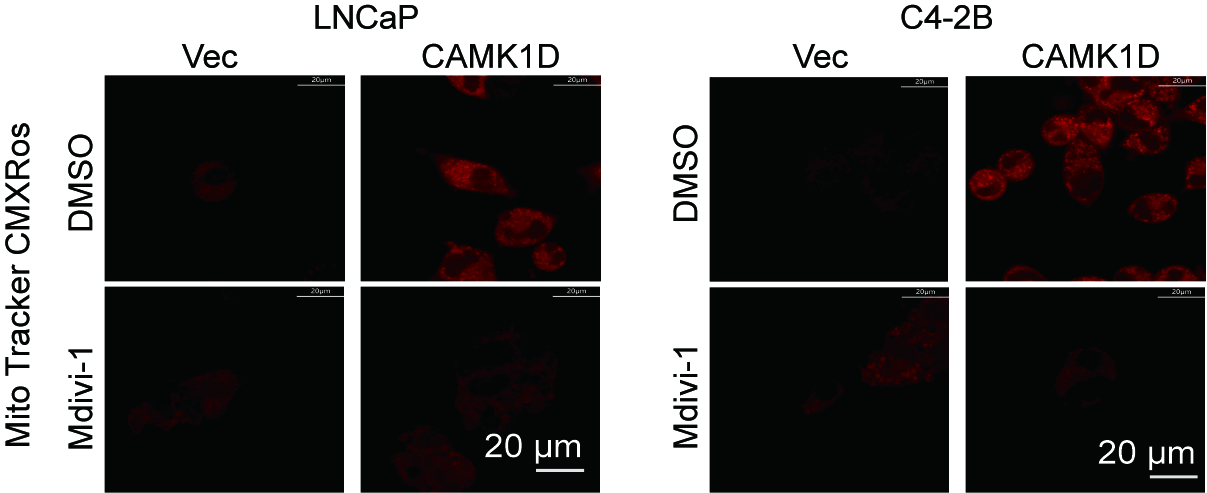


**Supplementary Fig. 6: Mdivi-1 effectively inhibits mitophagy in PCa cells.** LNCaP and C4-2B cells overexpressing CAMK1D were treated with either DMSO or Mdivi-1 for 24 hours, and mitochondrial fluorescence was examined using Mito Tracker staining under confocal microscopy.


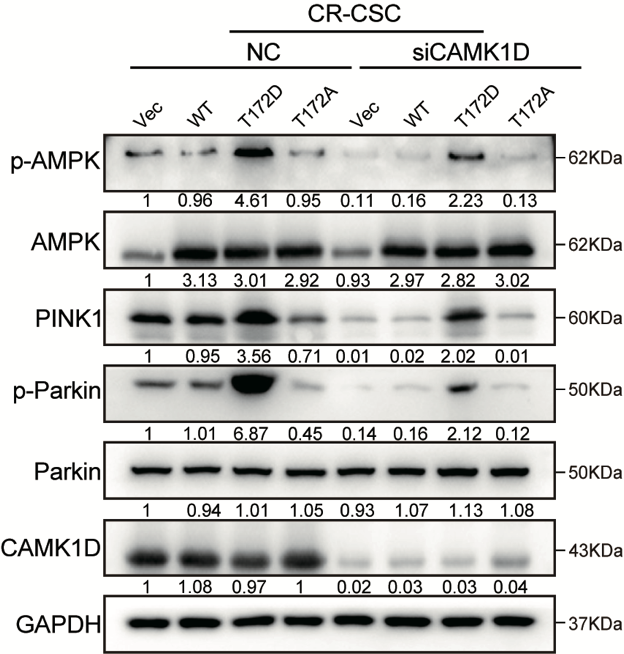


**Supplementary Fig. 7: CAMK1D-mediated PINK1/Parkin-dependent mitophagy is dependent on AMPK phosphorylation at Thr172.** In CR-CSC cells, either the full-length AMPK plasmid or AMPK Thr172 mutants-T172D to mimic phosphorylation and T172A to dominant-negative mutant, Parkin and PINK1 expression were then examined. The relative expression levels of target proteins were calculated as the ratio of the grayscale intensity of the target band to that of the GAPDH band.


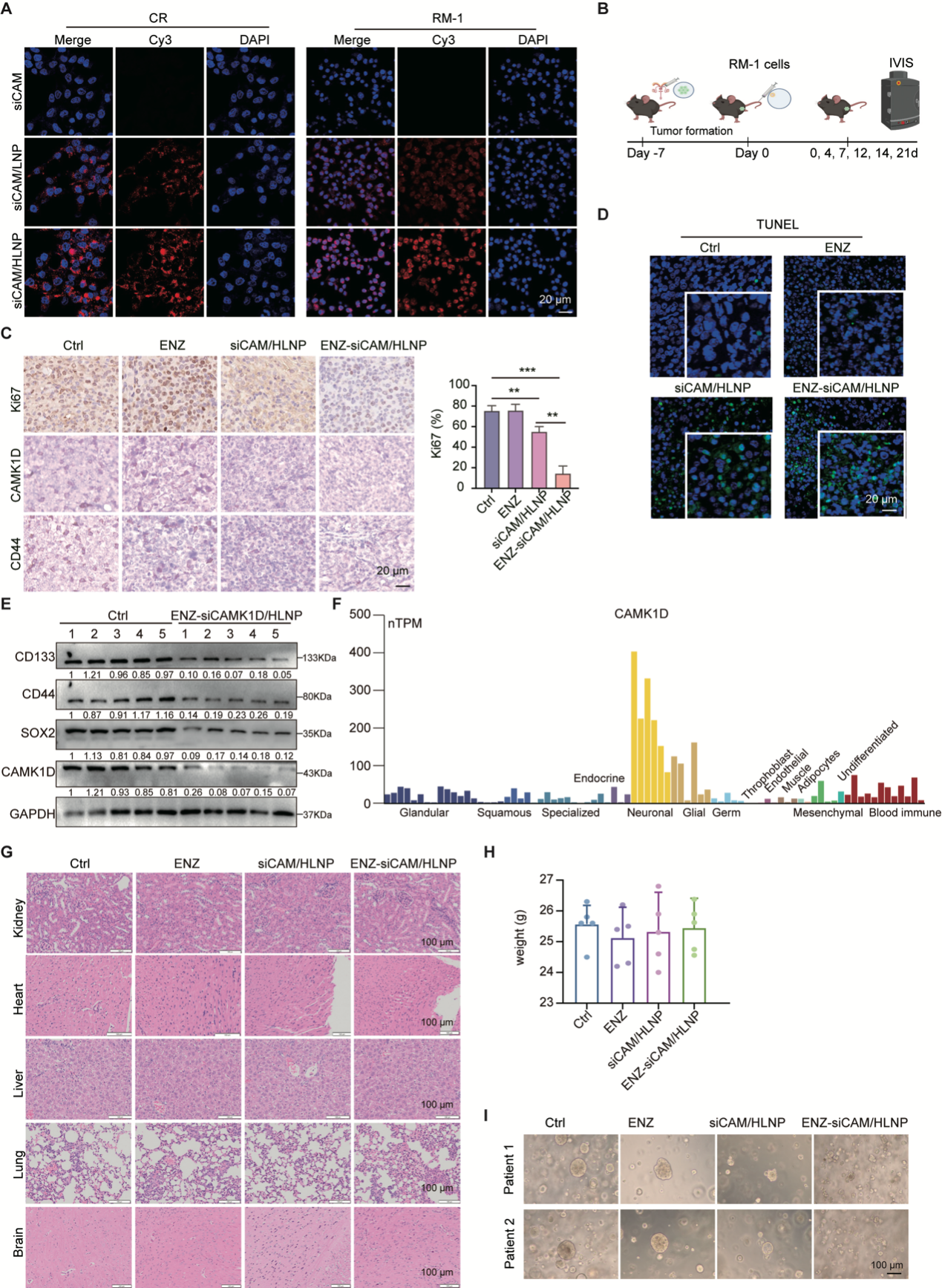


**Supplementary Fig. 8** **A CD44-targeted nanoplatform delivering siCAMK1D suppresses ENZR prostate cancer growth *in vitro* and *in vivo.*** **A** Cellular uptake of HLNP or LNP in enzalutamide-resistant PCa cells, assessed by confocal microscopy. siCAMK1D was labeled with Cy3. Scale bar: 20 µm. **B** Schematic diagram of the animal experiment design. **C** Ki67, CAMK1D, and CD44 expression analyzed by immunohistochemistry assay. **D** TUNEL staining results. **E** Western blot analysis of stemness-related markers in mouse tumor tissues. The relative expression levels of target proteins were calculated as the ratio of the grayscale intensity of the target band to that of the GAPDH band. **F** CAMK1D expression levels across different tissues. **G** Representative images of H&E staining to examine the toxicity of HLNP *in vivo* using lung, liver, heart, and kidney sections after necropsy. Scale bars, 100 μm. **H** Statistical chart of mouse body weight in different groups. **I** Representative images of two organoid samples with indicated treatments.
