# Supplementary figures and images for: CAMK1D activates AMPK/PINK1/Parkin-dependent mitophagy to promote enzalutamide resistance in prostate cancer
Source: Cell Death Dis. 2025 Dec 19;17(1):113. doi: 10.1038/s41419-025-08342-0 (PMC12847848; doi:10.1038/s41419-025-08342-0)

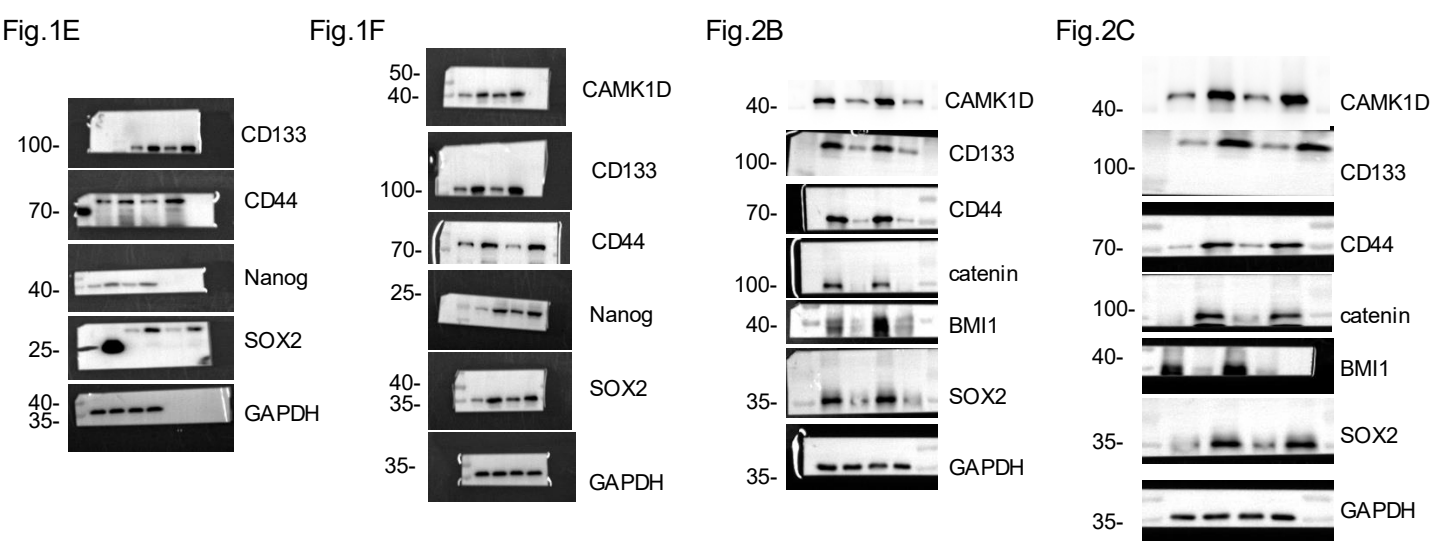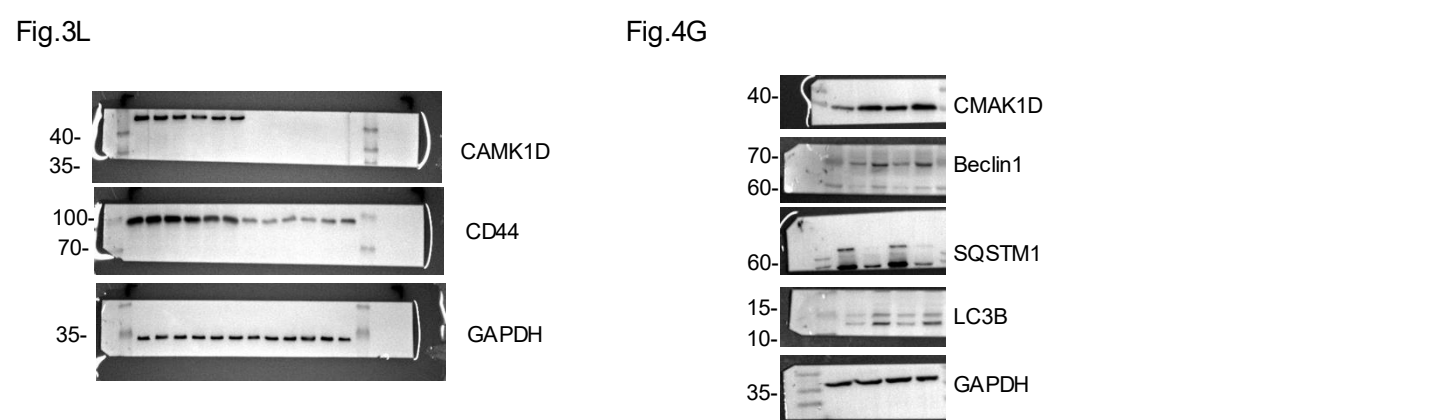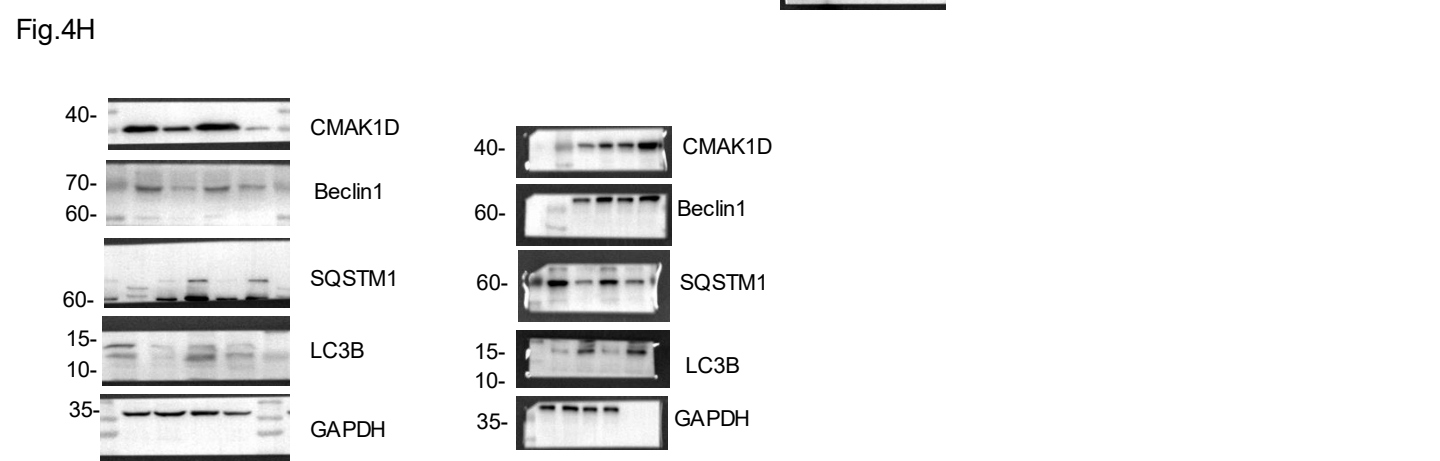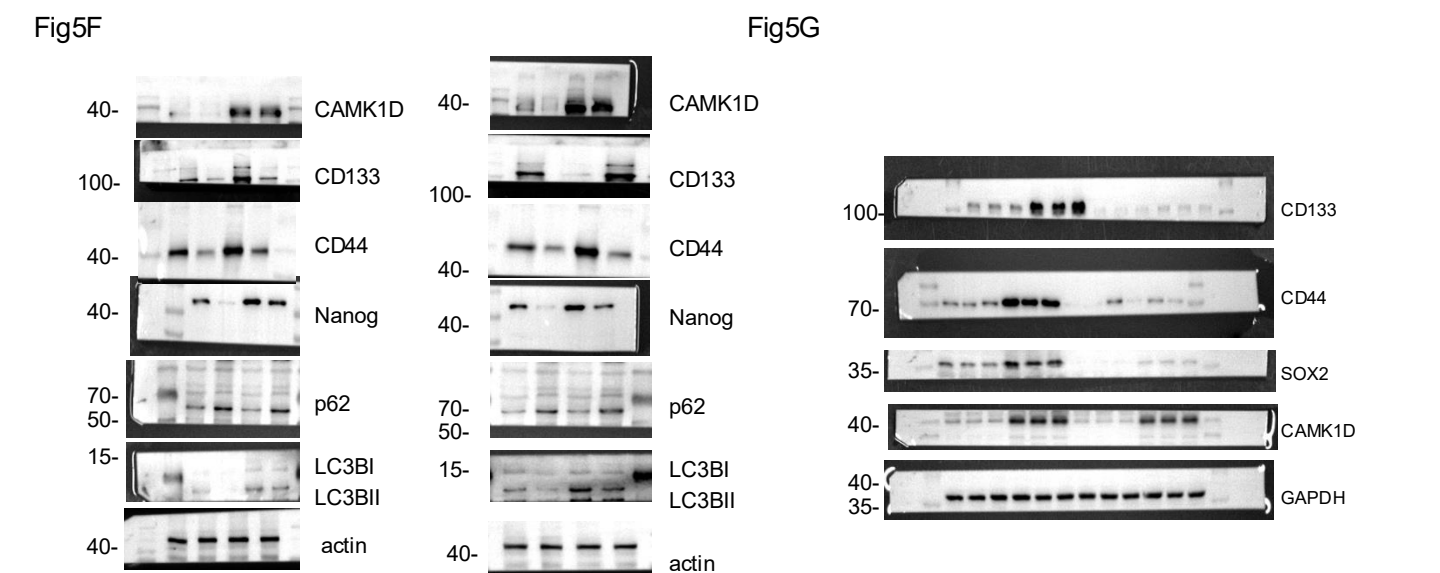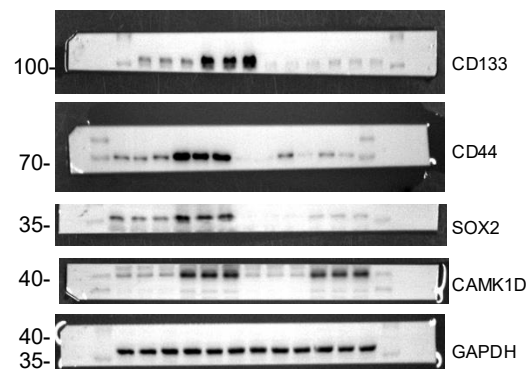

Fig. 6A

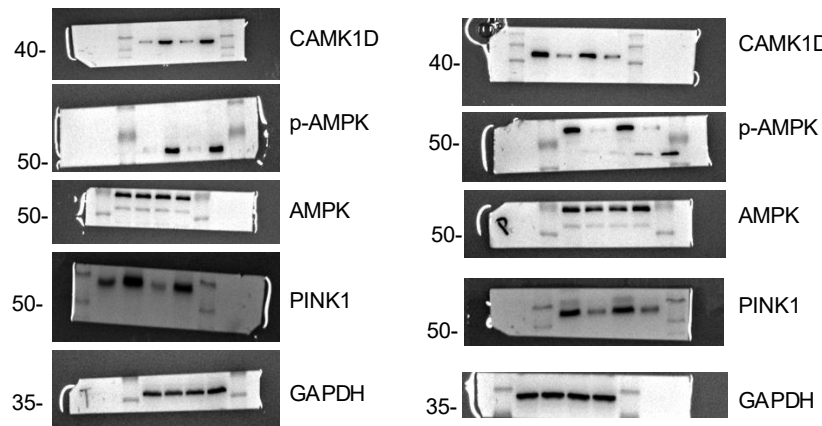

Fig.6B

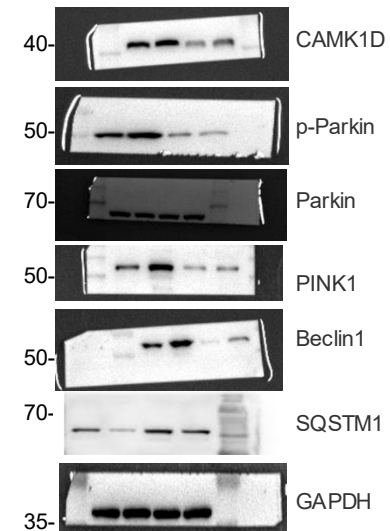

Fig.6B

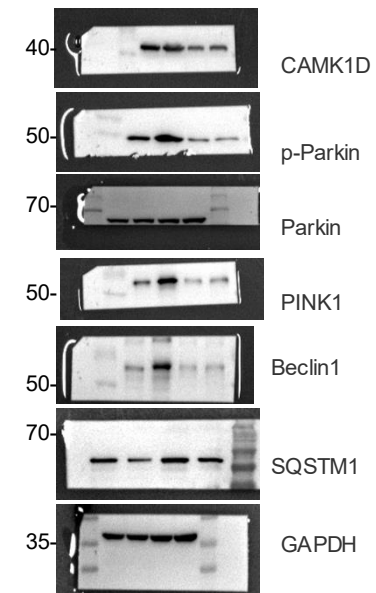

Fig.6C

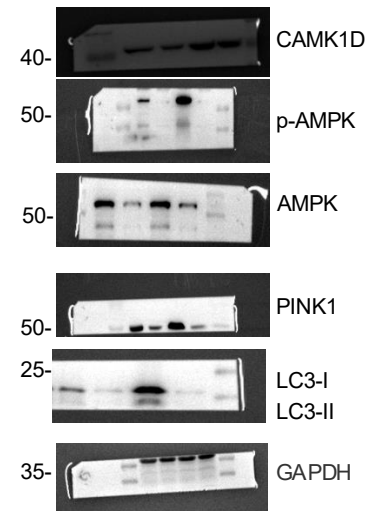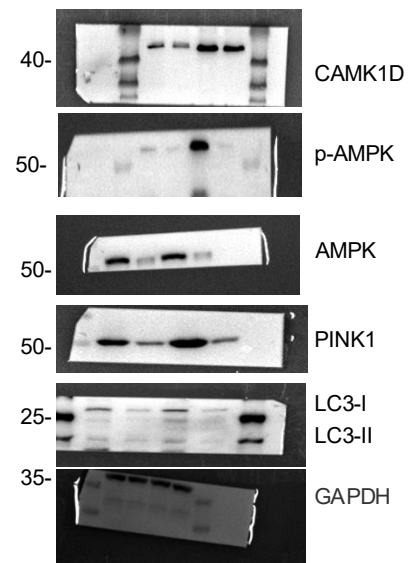

Fig. 6D

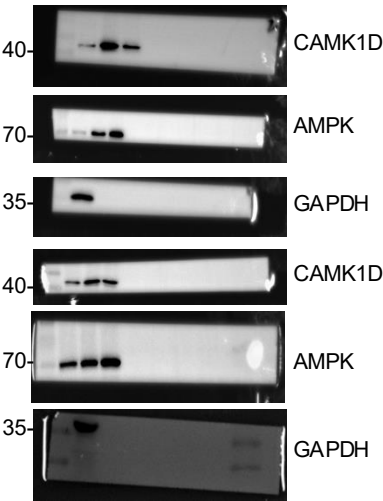

Fig. 6F

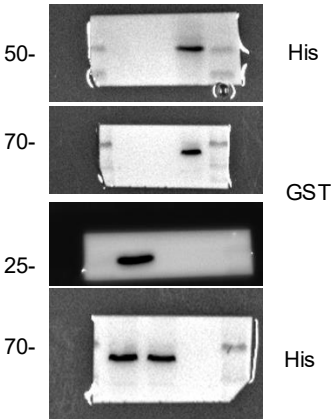

Fig. 6G

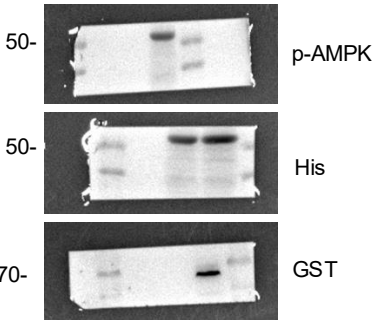

Fig.6I

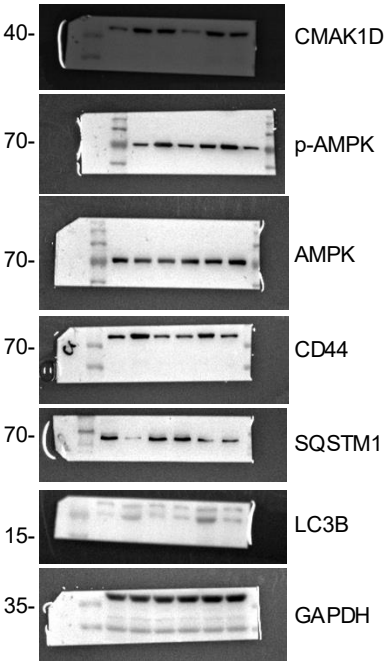

Fig.7F

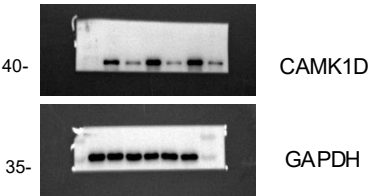

SFig.2A

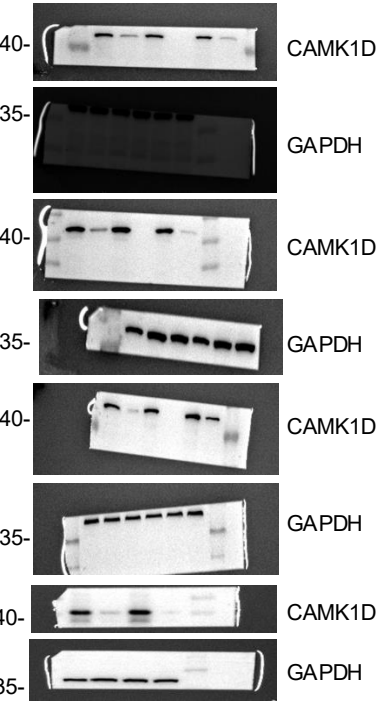

SFig.3D

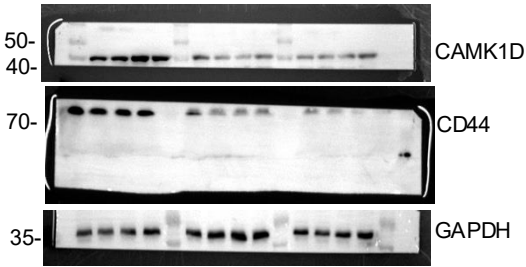

SFig.4C

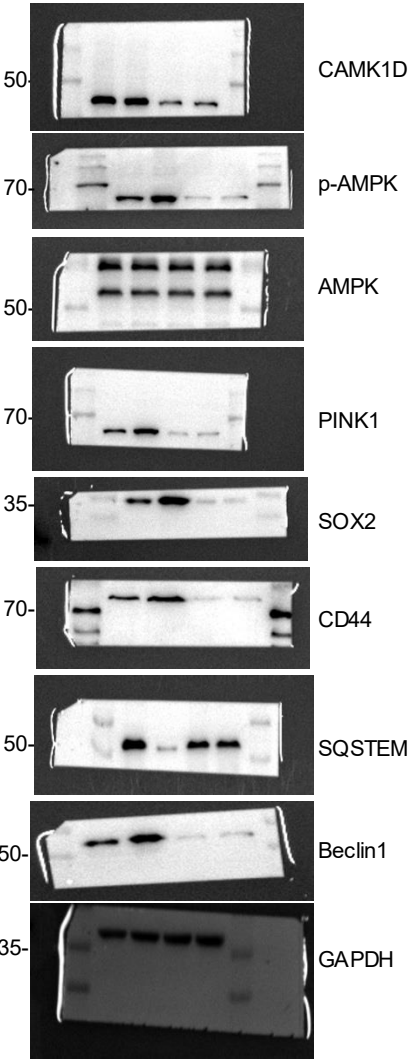

SFig.4D

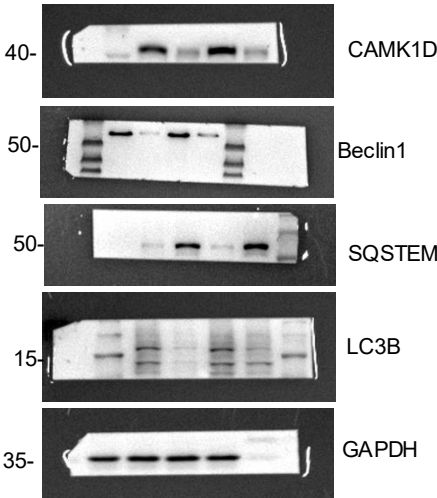

SFig.7

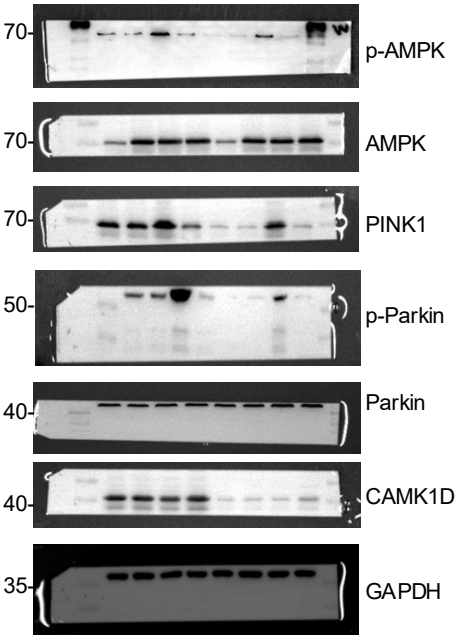

SFig.8E

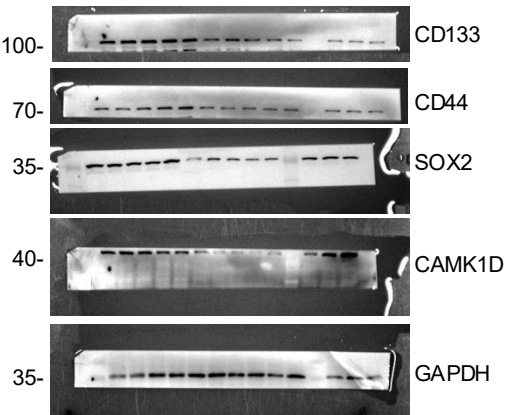

Supplement: Supplementary file 4 — Original Western blots [file 41419_2025_8342_MOESM4_ESM.pdf]
